# Knowledge, attitudes and practices about air pollution and its health effects in 6th to 11th-grade students in Colombia: a cross-sectional study

**DOI:** 10.3389/fpubh.2024.1390780

**Published:** 2024-06-19

**Authors:** Diana Marín, Nicolás Calle, Valentina Arango, Paulina Betancur, Manuela Pérez, Luz Yaneth Orozco, Beatriz Marín-Ochoa, Juan Carlos Ceballos, Lucelly López, Zulma Vanessa Rueda

**Affiliations:** ^1^School of Medicine, Universidad Pontificia Bolivariana, Medellín, Colombia; ^2^Faculty of Social Communication and Journalism, Universidad Pontificia Bolivariana, Medellín, Colombia; ^3^Department of Medical Microbiology and Infectious Diseases, University of Manitoba, Winnipeg, MB, Canada

**Keywords:** air pollution, environment and public health, awareness, attitudes and practices, knowledge, environmental health education, children, adolescent

## Abstract

**Introduction:**

Globally, air pollution is the leading environmental cause of disease and premature death. Raising awareness through environmental education and adequate communication on air quality could reduce the adverse effects. We aimed to assess the knowledge, attitudes, and practices (KAP) regarding air pollution and health and determine the factors associated with these KAP in children and adolescents.

**Methods:**

In 2019–2020, a cross-sectional study was conducted on 6th–11th grade high school students in five municipalities in Colombia. Variables collected included: age, sex, private or public school, any medical history, emergency room visits due to respiratory symptoms in the last year, and whether students played sports. The main exposure was the School Environmental Project. The outcomes were the KAP scale [0% (the lowest score) to 100% (the highest score)]. The factors associated with KAP levels were evaluated with independent mixed regressions due to the multilevel structure of the study (level 1: student; level 2: school), and the exponential coefficients (95% confidence interval-CI) were reported.

**Results:**

Among 1,676 students included, 53.8% were females. The median knowledge score about air pollution and its health effects was 33.8% (IQR: 24.0–44.9), 38.6% knew the air quality index, 30.9% knew the air quality alerts that occurred twice a year in these municipalities and 5.3% had high self-perceived knowledge. Positive attitudes, pro-environmental practices, being female, grade level, attending a private school, having respiratory diseases, and the school environmental project importance were associated with higher knowledge scores. The median attitudes score was 78.6% (IQR: 71.4–92.9). Pro-environmental attitudes were associated with knowledge-increasing, being female, attending a private school, and the school environmental project. The median pro-environmental practices score was 28.6% (IQR: 28.6–42.9). During air quality alerts, 11.6% had worn masks, 19% had reduced the opening time of windows and 15.9% avoided leaving home. Pro-environmental practices were associated with knowledge-increasing and attitudes-increasing, and lower practices with higher grade levels, visiting a doctor in the last year, and practicing sports.

**Discussion:**

Children and adolescents have low knowledge scores and inadequate pro-environmental practices scores regarding air pollution. However, they demonstrate positive attitudes towards alternative solutions and express important concerns about the planet’s future.

## 1 Introduction

Globally, air pollution is the leading environmental cause of disease and premature death, and a major contributor to climate change. According to the World Health Organization (WHO), 99% of the world’s population lives in polluted air, and 2.83 million deaths (10.95%) among women and 3.62 million deaths (11.81%) among men have been attributed to air pollution (1). In addition to health concerns, air pollution has economic implications and influences people’s compliance with sustainable development. A 1% increase in air pollution over the preceding three decades has resulted in a 10% increase in healthcare expenses, approximately $8.1 trillion in 2019, and 6.1% of the world’s gross domestic product (GDP) (2).

Although air pollution is a global problem, it is known that 79.3% of deaths attributed to this environmental factor occur in low-and middle-income countries (LMICs) (1), and less than 1% of those cities have air quality that meets WHO standards. The economic burden of air pollution exposure accounts for 9% of GDP in LMICs compared to 2.8% of GDP in high-income countries (HICs), demonstrating the environmental inequalities among people living in and experiencing poverty (3). People experiencing poverty are more likely to live in more polluted environments and to experience declines in economic productivity due to absences from work associated with environmental-related diseases, and in children, up to 43.1% of school absences are due to exposure to PM_2.5_ (4).

Reducing air pollution would have a significant impact on the health and well-being of children and adolescents (5–8). It is known that in children under 14 years of age, the primary risk factors associated with mortality are environmental, with air pollution (both outdoor and indoor) and high temperatures being the most significant factors (1). In addition, acute respiratory infections (ARI) are one of the primary causes of death in children; it has been reported that almost 26% of ARI deaths in children aged 5 to 14 are attributable to household air pollution (9).

Among the strategies that could potentially help reduce the adverse effects of air pollution are raising awareness through citizen science projects, adequate risk communication on air quality, and environmental education (10–12). It would have an important impact if environmental agencies formulated citizen science projects to improve and support their existing efforts of outreach and communication (13) and that include perceptions, needs, and values of the target population (14). The U.S. Environmental Protection Agency (EPA) has shown the effectiveness of citizen science projects in properly communicating risk, thereby increasing environmental awareness, and promoting behaviors that reduce exposure to wildfire smoke (12–15).

The relationship between environmental knowledge, pro-environmental attitudes, and practices is complex, and several theories have been proposed to explain the gap between possessing environmental knowledge and exhibiting pro-environmental behavior (16). Some research suggests that educational level could positively influence the level of knowledge about environmental problems and their causes (17, 18) but may not be directly correlated with pro-environmental attitudes (16). It also has been proposed that knowledge influences attitudes and values but does not have a direct impact on pro-environmental practices (16). In addition, being aware of air quality alerts, based on the Air Quality Index (AQI) which contains information about the potential health effects of air pollution, has been linked to changes in practices to reduce exposure in vulnerable individuals with pre-existing diseases (14, 18, 19). The relationship between attitudes and behavior has been more controversial (13); some studies indicate a positive relationship between high pro-environmental attitudes and more ecologically responsible behaviors (20), however, others have shown a non-significant effect (21). Factors contributing to the gap between attitudes and practices include gender, educational level, economic, social, and cultural factors, motivation, attitudes, and values learned within the family, neighborhood, and schools (16, 21, 22).

Given that some aspects of environmental education are taught in elementary and high schools and because a high burden of disease in young people is caused by the environment, it is crucial to understand the knowledge, attitudes, and practices (KAP) that young people have regarding this subject. The majority of KAP studies have been conducted in HICs (18, 23–27), mostly China, the U.S., and a few in low-income countries (LICs) (28, 29) and upper-middle-income countries (21). These studies (23–26, 28, 29) have shown differences in the KAP between countries. The knowledge regarding air composition, causes, and consequences is higher in HICs compared to low-and middle-income countries (LMICs), but the attitudes are different between countries no matter their income. To our knowledge, there are no known studies on KAP about air pollution among high-school students in Latin American or Caribbean countries.

Medellin is the second-largest city in Colombia, with 5% of the country’s population residing there. It is one of the most important centers for industrial, textile, and car development in Latin America (30). During the previous decade, the average yearly levels of PM_2.5_ have fluctuated between 20 g/m^3^ and 33 g/m^3^ (31), which is four times the WHO-recommended daily values (PM_2.5_ annual: 5 g/m^3^) (32). While national strategies have been implemented to strengthen environmental education in academic institutions through the development and implementation of school environmental projects (PRAE) (33), and local environmental entities have strengthened risk communication (34), it is unknown if these strategies, adopted and implemented by schools, are associated with the increasing KAP levels in children and adolescents in the city. Therefore, we aimed to determine the KAP levels about air pollution among children and adolescents in public and private schools in five municipalities in Colombia, as well as identify factors associated with KAP levels.

## 2 Materials and methods

### 2.1 Study design

We conducted a cross-sectional study between October 2019 and January 2020, prior to the COVID-19 pandemic. Medellin and four other nearby municipalities (Bello, Barbosa, Itagui, Caldas) were chosen because they had environmental emergencies in the past 5 years, defined as levels of PM_2.5_ that exceeded the air quality national regulations (PM_2.5_ 24-h: 37 g/m^3^) (35). The same environmental authority is in charge of these five municipalities, and when there is an environmental emergency with the air quality (usually in March and October of each year), different steps are taken to protect the health of children, the older adult, pregnant women, and people with pre-existing respiratory diseases who are most likely to be affected by air pollution (36).

### 2.2 Study population

Sixth to eleventh grade students were chosen from public (*n* = 5) and private (*n* = 3) schools in these municipalities. In Colombia, secondary students are between 11 and 16 years of age. On the day of the survey, all students in the randomly selected group were invited to participate, and those who voluntarily agreed with and signed the consent form were included. Participants with psychological or psychiatric conditions that limited their ability to provide informed consent and respond to the survey were excluded from the study.

### 2.3 Sample size

Our main research question was what are the knowledge, attitudes, and practices about air pollution among 6th to 11th grade students? In addition, our secondary research question was what is the relationship between knowledge, attitudes, and practices about air pollution, and what other factors influence the KAP scores in this population?

Our primary outcome for sample size calculation was focused on the main research question. Based on previously published research, we used an overall prevalence of correct knowledge about the biological effects of air pollution of 57.7% (37). Using this prevalence, our assumptions were an absolute precision of 3.5%, a 95% confidence level, and a design effect of 2 to account by cluster effect (school), we calculated a sample size of 1,548 children. The sample was increased by 8%, for a total sample of 1,676, to compensate for missing information in the questionnaires. The sample size calculation and the random selection of groups were carried out using Epidat 4.2 (Galicia, Spain). We randomly selected one group per grade in each school using the lists provided by each school.

### 2.4 Data collection

The anonymous KAP questionnaire was self-administered. We did not request names, identification numbers, or emails from the students. Four researchers received prior training and recruited the students. Each KAP questionnaire was distributed in each selected classroom, and we answered any questions that students may have. After we explained the purpose of the study and obtained the written consent forms, students who agreed to participate filled out the questionnaire, and then researchers reviewed its completeness and potential inconsistencies. In total, the process took 40 min in each group. A second quality check of each questionnaire was carried out before typing the information into the database.

### 2.5 Variables

The following aspects were considered as variables:

#### 2.5.1 Sociodemographic characteristics and personal history

Age, sex at birth (female, male), type of school (public or private), school grade (6th – 11th), any medical history (none, respiratory and other disease), visits to a doctor or emergency room in the last year for respiratory symptoms, and whether the student plays sports.

#### 2.5.2 Main exposure

School Environmental Project. Colombia has a legal framework that requires schools to develop school environmental projects (PRAE is the acronym in Spanish ‘Proyecto Ambiental Escolar’) to enhance environmental awareness and increase environmental education. This national law was developed jointly by the Ministry of Environment and the Ministry of Education (33). It includes local or regional environmental issues analyzed, studied, and addressed at the schools by students and teachers. The requirements to be an important PRAE include that the development of the project encompasses an environmental analysis of the context in which each school is located, and a participatory process with the entire school community and the general population. Additionally, the main issue(s) prioritized in the school environmental project should be incorporated into the curriculum and school facilities for environmental improvement by the school members. The implementation of each project aims to enhance environmental awareness and increase the environmental education of students.

We evaluated the importance of the project for each school through an interview with the school environmental project coordinator, a focus group with key students participating in the school environmental project, and a documented analysis of each project. Supplementary Tables S1, S2 include the indicators and scores used to assess the importance of each project. In summary, four dimensions were evaluated with 27 indicators. Each indicator had a score between 0 and 3, with 3 representing the highest level of importance. The overall importance score was the sum of the 27 indicators. The maximum score for a project was 81 points, which meant an impact grade of 100%. The maximum grade meant the school environmental project had a high importance in the development and implementation. The score quartiles were calculated as low importance: ≤60%, median importance: 61–70%, and high importance: ≥71%. The importance scores (low, median and high) of the school environmental project were included in the KAP multivariate analyses.

The findings of the school environmental project evaluation, as well as videos made by the students inviting adults to take care of the environment and infographics illustrating the findings of this study, are available on the website https://escuelaenelmapa.com/.

#### 2.5.3 Knowledge variables

The student’s knowledge was evaluated through 35 items. Thirty-two evaluated the composition of clean and unpolluted air, the air pollution sources, the biological and physiological effects of air pollution, acid rain, and the greenhouse effect. A Likert scale of five options was used. If the questions were formulated in a positive sense or were true, for example, “There is nitrogen in the clean and unpolluted air,” the options *“I’m sure this is right”* and *“I think this is correct”* were considered correct knowledge and were coded as 1. The choices *“I do not know about this,” “I believe this is wrong,”* and *“I am sure that this is incorrect”* were coded as 0 and considered incorrect or no knowledge. For items formulated in a negative or false sense, for example, “If the air looks clear, it’s not polluted,” the two options on the Likert scale, *“I think this is wrong”* and *“I’m sure this is incorrect,”* were considered correct knowledge and coded as 1.

Additionally, students were asked if they knew what the Air Quality Index (AQI) was (Completely known, partially known, and not known at all) and how much they knew that because their city is surrounded by mountains and has a large number of vehicles and motorcycles, there are two seasons of the year when clouds prevent air pollutants from escaping, causing air quality alerts (Completely known, partially known, and not known at all). One last question was the self-perception of knowledge about air pollution (I know a lot, I know very little, I do not know anything).

#### 2.5.4 Attitudes variables

The students’ attitudes were assessed with 14 items. Twelve of these items were designed to identify their feeling about whether there should be greater emphasis on various actions to reduce air pollution, including education, responsibilities, taxes, and legislation, among themselves and their friends, factories, transport companies, and the general population. A Likert scale of five options was used, and the *“I agree”* and *“I very much disagree”* options were coded as 1 to indicate a positive attitude towards the action to reduce air pollution, and the options *“I do not agree or disagree,” “I do not agree,”* and *“I very much disagree”* indicated a negative attitude towards the action to reduce air pollution and were coded as 0.

The other two questions were how students felt about air pollution (“Very worried,” “A little worried” and “I do not feel worried at all”) and how they felt when someone approached them to talk about environmental care (“It does not interest me,” “It seems boring,” “I feel I’m too young to help the environment,” “I’m more interested in the subject and wonder how I can help,” and “I find out more about that subject with my parents, teachers, or social networks”).

#### 2.5.5 Practices variables

For the practices section, a literature review was conducted, and seven questions were selected to include in this section. The validity of the questionnaire was established through expert content validation, and internal consistency was determined through the Cronbach alpha [value of 0.80 (95% confidence interval-CI: 0.782–0.821)]. A pilot study was conducted to adjust the language, content, readability, and order of the questions.

The environmental practices’ component evaluated how students search for and get information about air pollution and what steps they take to protect their health from the effects of air pollution. In addition, we asked if they checked, daily, the air quality index (AQI) through the free SIATA app,^1^ developed by the environmental authority of these municipalities.

We also collected the following types of sources that students used to learn about air pollution and air quality alerts in the municipality (internet, social media, news, newspapers, friends, teachers, parents, and school). Cancellation of sports classes due to air quality alerts, if they avoided going out from home or used a facemask to protect themselves from air pollution when they left their houses, and if they implemented additional measures at home such as closing windows and doors while air quality alerts occurred.

The KAP questionnaire included 69 items in total (See Supplementary data and data availability statement).

#### 2.5.6 Outcomes

Each component was transformed into a score ranging from 0 to 100% to facilitate interpretation and comparison between them.

##### 2.5.6.1 Knowledge scale

A knowledge scale was built based on 35 items coded as 1 or 0 and was transformed into a score from 0 to 100%, with 0% meaning the lowest level and 100% meaning the highest level of knowledge. As a proxy for specific knowledge regarding particulate matter, the two knowledge questions about the AQI and air quality alerts in the cities along with self-perceived knowledge were explored as outcomes.

##### 2.5.6.2 Attitudes scale

The attitudes scale was based on the 14 items coded as 1 and 0 and was transformed into a score from 0 to 100%, with 100% being the highest attitude value towards air pollution.

##### 2.5.6.3 Practices scale

For the practices scale, the seven evaluated items were coded as 0 and 1 and were used to construct the scale of practices ranging from 0 to 100%, with 0% being the lowest level of informative and preventive practices about the effects of air pollution.

### 2.6 Statistical analysis

A descriptive analysis was done for the sociodemographic characteristics and the KAP scales. The age and scales of knowledge, attitudes, and practices were reported using the median and interquartile range (IQR: Q1, Q3) by type of school (public or private). To determine differences by type of school, the Chi square test was used for the qualitative variables, and for the quantitative variables the Mann–Whitney’s U test was used. The description of the KAP scale by independent variables was performed using violin plots including boxes and whiskers.

To identify the factors associated with knowledge, attitudes, or practices about air pollution, we checked the distribution and skewness of each outcome. Verification of the assumptions can be found in Supplementary Figures S1. The KAP scales’ results followed an approximately normal distribution for the knowledge scale, a negative asymmetry for the attitudes scale, and a positive asymmetry for the practices scale.

Due to the multilevel structure of the study (level 1: student; level 2: school), mixed regressions were conducted considering the distributions of the KAP scores as follows, a linear mixed model for knowledge, a negative binomial mixed model for attitudes, and a gamma mixed model for practices. In each model, a log link function was used, with an exchangeable correlation structure, and standard errors were adjusted for the school cluster. To facilitate the interpretation of the coefficients, they were exponentiated, and the prevalence rate ratio was reported with 95% confidence intervals (95%CI). The main exposure was the school environmental project’s importance which was included in all models. Independent variables were sex, grade level, type of school, the presence of respiratory diseases, visits to a doctor in the last year and to practice sports. The outcomes were: Scores of knowledge (model 1), attitudes (model 2), and practices (model 3). A bilateral *p*-value <0.05 was considered significant. The analysis was performed in SPSS 24.0, Stata 15.0 and Jamovi 2.4.

### 2.7 Ethics approval

This research was approved by the Health Research Ethics of the Pontifical Bolivarian University on September 30, 2019 (Approval N ° 18–2019) and the directors of each institution. A printed letter outlining the study was sent to all parents and students before the data collection. All students signed a written consent form in the presence of two witnesses, and they were informed that their participation was voluntary and could be withdrawn at any moment. We used anonymous questionnaires to reassure all students that their individual questionnaire scores would not be shared with any school member and would not affect their academic performance. In addition, we clearly explained that they could stop answering the questionnaire at any time.

## 3 Results

There were 1,676 surveys with 15 excluded because of incomplete information. Of the 1,661 students included in the analysis, the median age was 14 years (IQR: 13, 16), with 1% being older than 17 years, 73.3% were between 6^th^ and 9^th^ grade, and 53.8% were female. The level of knowledge in the composition of clean air, and on acid rain and the greenhouse effect was higher in private schools than in public schools (*p* < 0.001) (Table 1).

**Table 1:** Sociodemographic characteristics and knowledge, attitudes, and environmental practices in elementary and high school students according to type of school in Colombia, 2019–2020.

Variable, *n* (%)	Private (*n* = 449)	Public (*n* = 1,212)	*p*-value	Total (*n* = 1,661)
	*n* (%)	*n* (%)		*n* (%)
Female sex	275 (61.9)	609 (50.8)	<0.01	884 (53.8)
Age in years, Me (Q1, Q3)	14 (13, 16)	14 (13, 16)	0.56	14 (13, 16)
Minimum - Maximum	10–21	11–19		10–21
Grade level			0.22	
6–7	175 (39.0)	455 (37.5)		630 (37.9)
8–9	168 (37.4)	420 (34.7)		588 (35.4)
10–11	106 (23.6)	337 (27.8)		443 (26.7)
Practice sports, total	449	1198		1647
Practice some sport	241 (53.7)	697 (58.2)	0.10	938 (57.0)
Sport >3 h/week	176 (73.0)	500 (72.8)	0.10	676 (72.8)
Medical history*				
None	344 (74.3)	962 (78.9)	0.05	1,306 (77.6)
Respiratory	55 (11.9)	110 (9.0)	0.09	165 (9.8)
Other disease	64 (13.8)	148 (12.1)	0.39	212 (12.6)
Visits to a doctor in the last year for respiratory symptoms	65 (14.5)	169 (14.1)	0.82	234 (14.2)
Knowledge of Air quality index, total	447	1187		1634
Completely known	24 (5.4)	62 (5.2)	0.23	86 (5.3)
Partially known	163 (36.5)	381 (32.1)		544 (33.3)
Not known at all	260 (58.2)	744 (62.7)		1,004 (61.4)
Knowledge of air quality alerts twice a year, total	445	1169		1614
Totally known	161 (36.2)	337 (28.8)	0.01	498 (30.9)
More or less known	182 (40.9)	556 (47.6)		738 (45.7)
I had no idea that it was happening.	102 (22.9)	276 (23.6)		378 (23.4)
Auto-perceived knowledge about air pollution, total	416	1070	0.02	1486
Much	49 (11.8)	145 (13.6)		194 (13.1)
Little	353 (84.9)	852 (79.6)		1,205 (81.1)
Nothing at all	14 (3.4)	73 (6.8)		87 (5.9)
Feelings about air pollution, total	433	1078		1511
Very worried	272 (62.8)	645 (59.8)	0.34	917 (60.7)
A little worried	151 (34.9)	395 (36.6)		546 (36.1)
I do not feel worried at all	10 (2.3)	38 (3.5)		48 (3.2)
Levels of KAP, Me (Q1, Q3)				
Clean air composition	36.4 (18.2, 45.5)	27.3 (18.2, 36.4)	<0.01	27.3 (18.2, 45.5)
Air pollution sources	66.7 (50.0, 83.3)	50.0 (33.3, 66.7)	<0.01	50.0 (33.3, 77.8)
Air pollution effects	55.6 (44.4, 77.8)	55.6 (33.3, 66.7)	0.01	55.6 (33.3, 50.0)
Acid rain and greenhouse effects	50.0 (33.3, 66.7)	33.3 (16.7, 50.0)	<0.01	33.3 (16.7, 50.0)
Knowledge	38.8 (29.9, 50.5)	31.8 (22.2, 42.3)	<0.01	33.8 (24.0, 44.9)
Attitudes	78.6 (71.4, 85.7)	78.6 (64.3, 92.9)	0.78	78.6 (71.4, 92.9)
Practices	42.9 (28.6, 42.9)	28.6 (28.6, 42.9)	<0.01	28.6 (28.6, 42.9)

Me, median; Q1, quartile 1; Q3, quartile 3; KAP, Knowledge, attitudes, practices. ^*^One student may report more than one diagnosed disease ^**^Visits for respiratory diseases.

Three out of five public schools included in the study were classified with the highest importance score (high importance), one was classified as medium, and one was classified as lower, as well as one of the private schools (low importance). None of the three private schools were classified in the high-importance category, as shown in Supplementary Table S3.

### 3.1 Knowledge

It was found that 87% of students self-perceived to have little or no knowledge about air pollution, with differences found by type of school (*p* = 0.02). The lowest scores were in the composition of clean and unpolluted air (27.3%) and acid rain and the greenhouse effect (33.3%), with higher knowledge levels in students from private schools (*p* < 0.001) (Table 1). A total of 5.3% of students were familiar with the AQI (Air Quality Index). The knowledge about air quality alerts twice a year was significantly lower among students in 6-7th grade (26.7%) compared to students in 8-9th grade (32.2%) and 10-11th grade (34.9%) (*p* < 0.001), see Supplementary Table S4.

**Figure 1:**
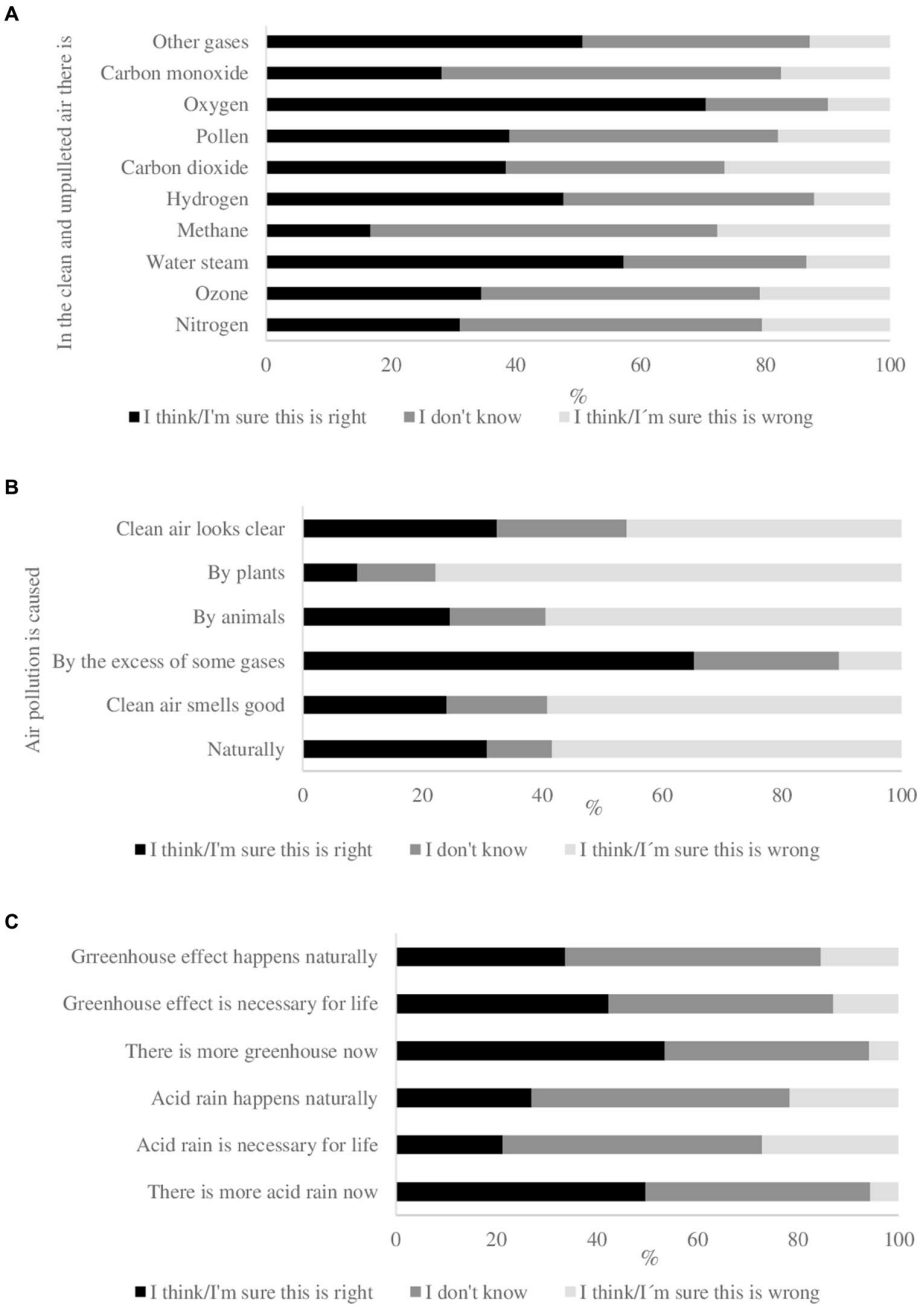
Answers among elementary and high school students in Colombia about knowledge in air pollution. **(A)** Composition of clean and unpolluted air, **(B)** Sources of air pollution, **(C)** Acid rain and greenhouse effect.

For the composition of clean and unpolluted air, the two components mostly identified were oxygen (70.5%) and water steam (57.3%), and only 31.1% identified nitrogen as a component of clean and unpolluted air, despite being the gas with the largest volume in the atmosphere (Figure 1A). It is worth noting that between 30 and 50% of students did not know whether the evaluated components were part of clean and unpolluted air.

**Figure 2:**
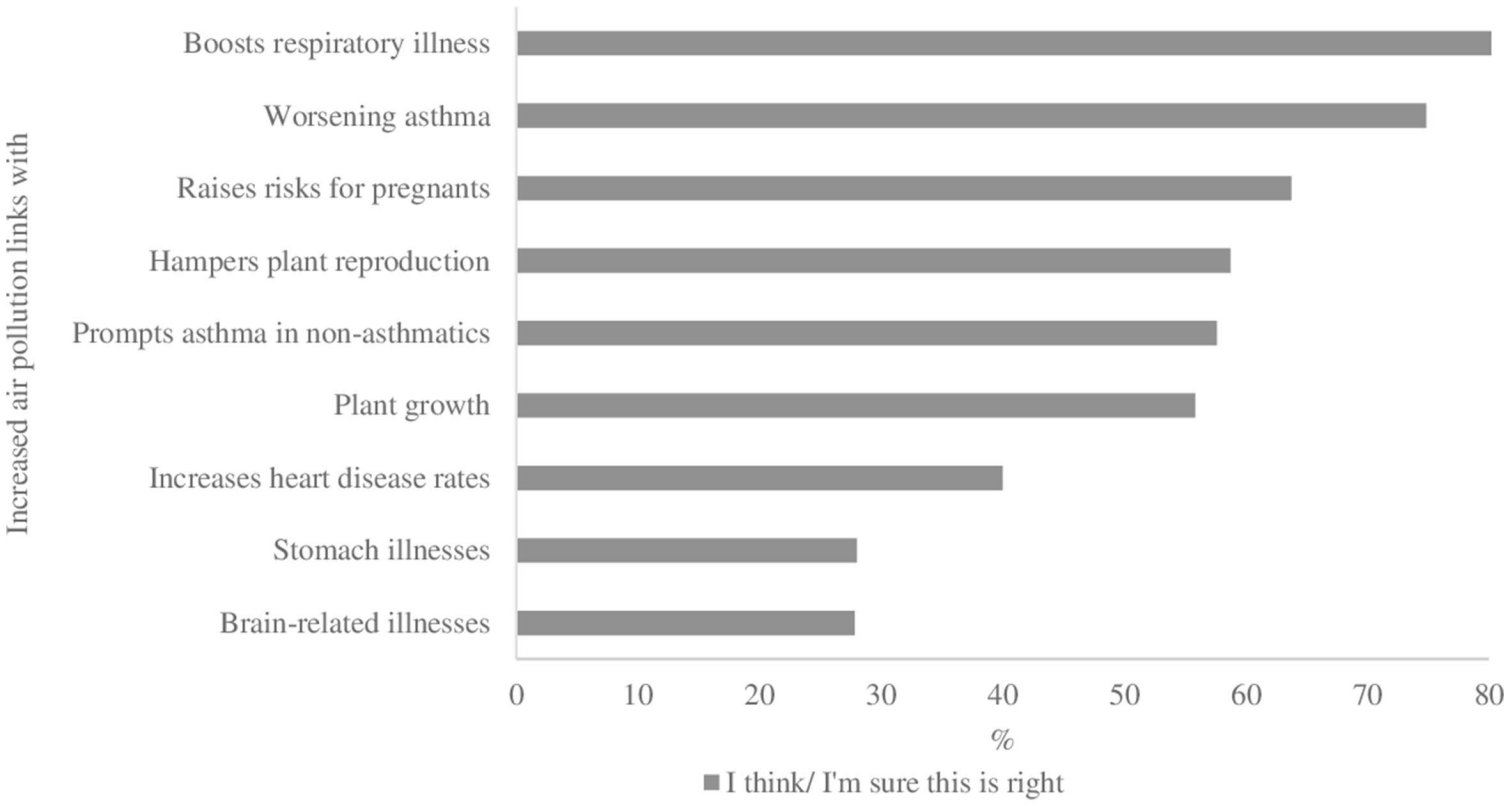
Knowledge of the effects related to the increase in air pollution among elementary and high school students in Colombia, 2019–2020.

The excess of any gas as the causative factor of air pollution was recognized by 65.4% of students, while 30.8% thought it happened naturally (Figure 1B). As for acid rain, despite being an anthropogenic phenomenon, 21.7% thought it was a natural phenomenon, and 33.7% recognized that the greenhouse effect is a natural event exacerbated by air pollution (Figure 1C). The development and exacerbation of respiratory diseases were reported as the main health effects related to increased air pollution (Figure 2).

### 3.2 Attitudes

**Figure 3:**
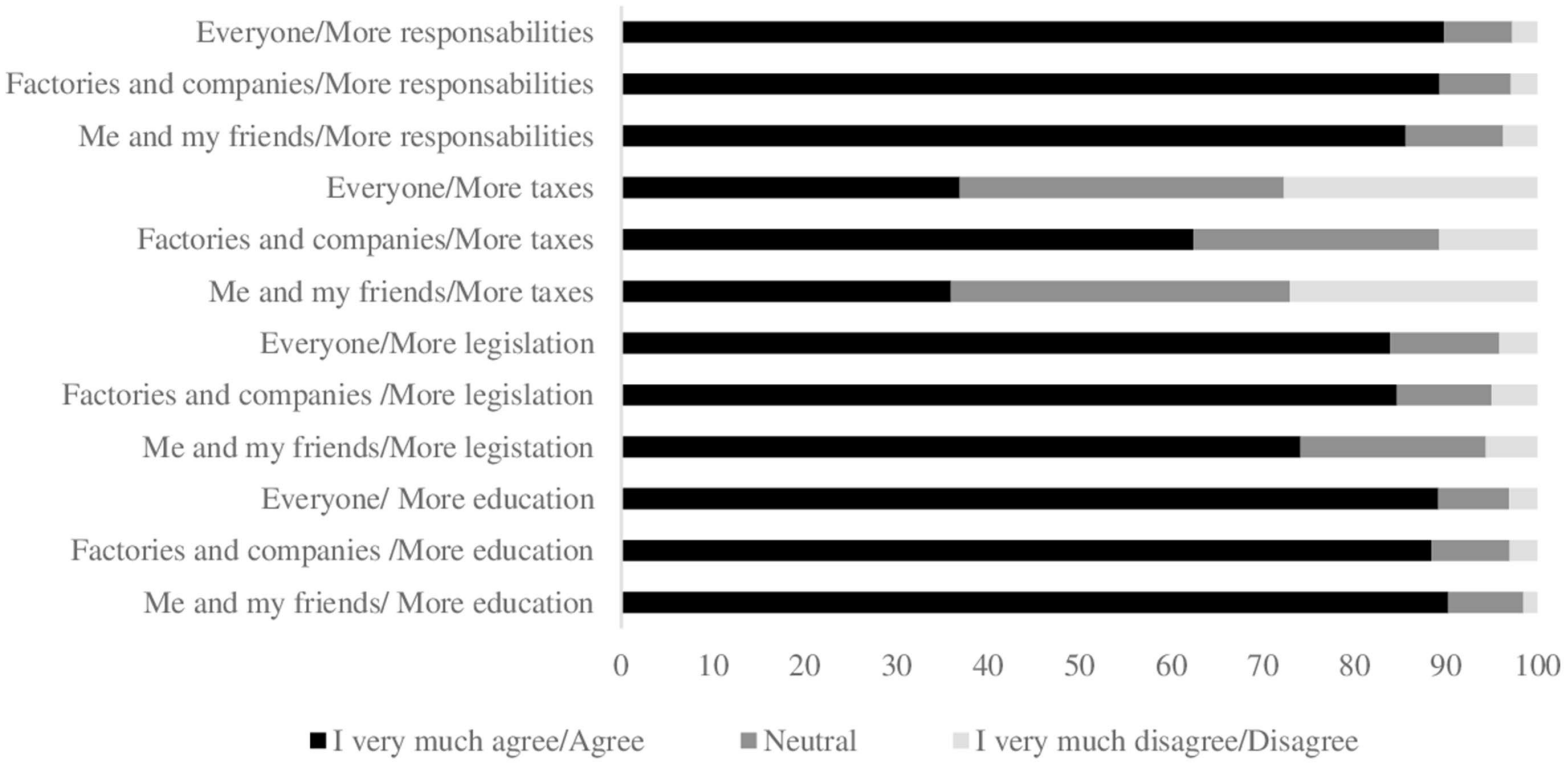
Students’ feelings about whether there should be greater emphasis on education, responsibilities, taxes, and legislation, among themselves and their friends, factories and transport companies, and the general population to reduce air pollution.

**Figure 4:**
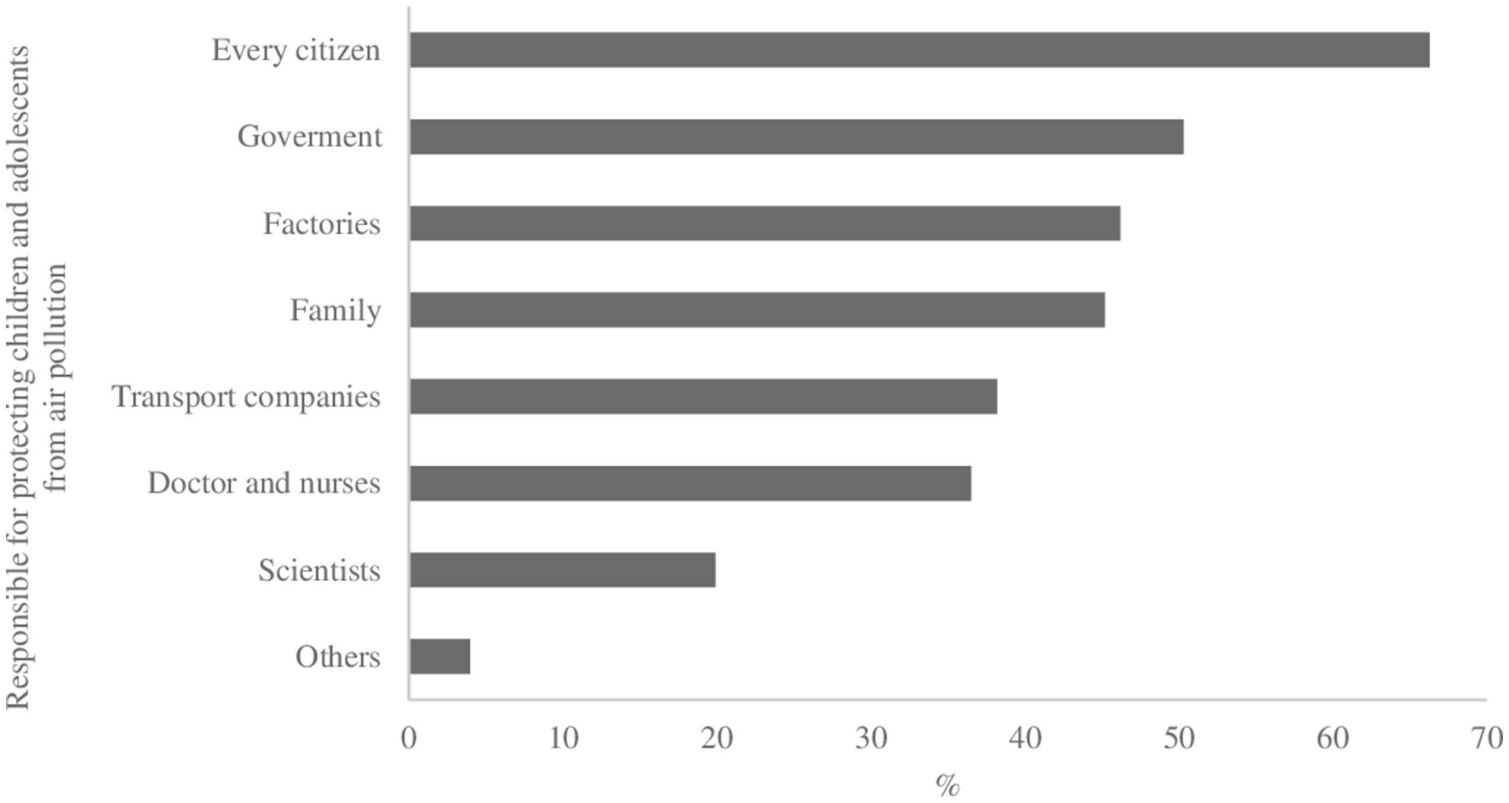
Students’ opinion on the main agent that should protect children and adolescents from air pollution in Colombia.

The median score on the attitudes scale was 78.6% (IQR: 71.4; 92.9) with no differences by type of school (*p* = 0.39) (Table 1). Nearly 90% of students had a positive attitude about implementing taxes to stop air pollution and that the entire population, including themselves, factories, and transport companies, should receive more environmental education. The attitude towards the general population paying taxes to stop or decrease air pollution tends to be negative, with 62.4% of students considering that it should be factories and transport companies who pay those taxes (Figure 3). Most students believed that every citizen (66.3%), government (50.3%), and industry (46.2%) were responsible for protecting children and adolescents from air pollution (Figure 4).

### 3.3 Practices

The median score on the practices scale was lower in students from public schools (*p* < 0.001) and the component with the lowest score on the KAP scale (Median: 28.6: RIC: 28.6, 42.9) (Table 1); 15.6% of those who knew AQI completely or partially also consulted it as a warning of air pollution health effects, with this practice being 4 times more frequent in students from public schools (*p* < 0.001) (Table 2). Students reported that the main source of information about air quality and air pollution reports comes from the news (45.5% of the time) and the internet (44.3% of the time). As for preventive measures, 58.5% never avoided leaving home due to air pollution, with no differences by type of school (*p* = 0.49), and 54.1% did not close windows and doors at home, with this practice being 1.56 times more common among students from private schools (*p* < 0.001) (Table 2).

**Table 2:** Practices regarding air pollution in elementary and high school students in Colombia, 2019–2020.

Variable, *n* (%)	Private (*n* = 449)	Public (*n* = 1,212)	*p*-value	Total (*n* = 1,661)
Verify daily air quality index, total^*^	185	423		608
Yes	9 (4.9)	86 (20.3)	<0.01	95 (15.6)
No	113 (61.1)	171 (40.4)		284 (46.7)
SIATA not known	63 (34.1)	166 (39.2)		229 (37.7)
Sources consulted to learn about air pollution, total/responses^**^	448/895	1190/2307		1638/3202
Internet	215 (47.9)	664 (54.8)	<0.01	879 (52.9)
Social networks	157 (35.0)	241 (19.9)		398 (24.0)
News	252 (56.1)	682 (56.3)		934 (56.2)
Teachers	74 (16.5)	296 (24.4)		370 (22.3)
Parents	123 (27.4)	269 (22.2)		392 (23.6)
None	74 (16.5)	155 (12.8)		229 (13.8)
Sources checked for air quality alerts, total/responses^**^	360/1062	1001/2968		1361/4030
Internet	193 (43.0)	542 (44.7)	<0.01	735 (44.3)
Social networks	139 (31.0)	337 (27.8)		476 (28.7)
News	193 (43.0)	563 (46.5)		756 (45.5)
Newspapers	70 (15.6)	228 (18.8)		298 (17.9)
Friends	64 (14.3)	224 (18.5)		288 (17.3)
Teachers	116 (25.8)	393 (32.4)		509 (30.6)
Parents	157 (35.0)	265 (21.9)		422 (25.4)
School	130 (29.0)	416 (34.3)		546 (32.9)
Cancellation of physical education classes due air quality alert, total	446	1186		1632
Sometimes	293 (65.7)	212 (17.9)	<0.01	505 (30.9)
Rarely	95 (21.3)	203 (17.1)		298 (18.3)
Never ever	58 (13.0)	771 (65.0)		829 (50.8)
Avoid leaving home due to air quality alert, total	447	1179		1626
Sometimes	63 (14.1)	195 (16.5)	0.49	258 (15.9)
Rarely	133 (29.8)	283 (24.0)		416 (25.6)
Never ever	251 (56.2)	701 (59.5)		952 (58.5)
Use of facemask as protection due air quality alert, total	446	1179		1625
Sometimes	34 (7.6)	155 (13.1)	<0.01	189 (11.6)
Rarely	60 (13.5)	180 (15.3)		240 (14.8)
Never ever	352 (78.9)	844 (71.6)		1,196 (73.6)
Reduce the opening time of windows and doors due air quality alert, total	446	1183		1629
Sometimes	60 (13.5)	250 (21.1)	<0.01	310 (19.0)
Rarely	112 (25.1)	325 (27.5)		437 (26.8)
Never ever	274 (61.4)	608 (51.4)		882 (54.1)

^*^Percentages are calculated based on those who knew AQI completely or partially. ^**^A student may answer more than one option. SIATA is the system for air quality alerts: https://siata.gov.co/siata_nuevo/.

### 3.4 KAP levels

The comparison of KAP scores according to the level of importance of the school environmental project and the independent variables can be found in Supplementary Table S5 and Supplementary Figures S2–S8. In general, schools with a median importance school environmental project had higher scores for knowledge (Median: 38.47; IQR: 29.80–50.25) and practices (42.86; 28.57–42.86) (*p* < 0.001), and there were no differences in the attitude score by the level of importance in the school environmental project (78.57; 71.43–92.86). Females had higher attitude scores, knowledge increased with the grade level, and students who reported having a respiratory illness and who reported visiting a doctor in the last year for a respiratory illness had higher knowledge and pro-environmental practices scores. The correlations among the three KAP scores were significant. See Supplementary Table S6.

### 3.5 Factors associated with KAP levels

Knowledge score increased by 0.5% (95% CI: 1.004–1.006) with an increase in attitude score, and by 0.1% (95% CI: 1.003–1.006) with an increase in practices score. An increase in the knowledge score led to a 0.5% increase in the attitude score (95% CI: 1.003–1.006) and a 0.7% increase in the practices score (95% CI: 1.005–1.009). The school environmental project of median importance was associated with a 6% increase in the knowledge score, while one of high importance was associated with a 6% reduction in the attitudes score. Being female was related to a 7% decrease in knowledge and a 10% increase in attitudes scores. It was found that as grade level increased, knowledge score steadily increased by up to 23%, while practices score decreased by 11%. Attending a private school was associated with higher knowledge score and lower attitudes score. Pro-environmental practices’ score decreased by 8% in children who consulted a doctor in the past year for respiratory symptoms and who practiced sports. Students with respiratory diseases had a 7% higher knowledge score (Table 3).

**Table 3:** Factors associated with knowledge, attitudes, and good practices against air pollution among elementary and high school students in Colombia, 2019–2020.

Variable	Knowledge			Attitudes			Practices		
	$e^{B}$	95%CI	*p*-value	$e^{B}$	95%CI	*p*-value	$e^{B}$	95%CI	*p*-value
Knowledge	–	–	–	1.005	1.004–1.006	<0.01	1.007	1.005–1.009	<0.01
Attitudes	1.005	1.004–1.006	<0.01	–	–	–	1.001	1.000–1.002	0.01
Practices	1.005	1.004–1.006	<0.01	1.001	1.000–1.002	0.05	–	–	–
Female sex	0.929	0.915–0.944	<0.01	1.103	1.088–1.118	<0.01	1.043	0.992–1.097	0.10
Grade level									
6–7	1			1			1		
8–9	1.111	1.019–1.211	0.02	1.038	0.985–1.094	0.16	0.901	0.864–0.939	<0.01
10–11	1.228	1.112–1.357	<0.01	1.042	0.984–1.103	0.16	0.893	0.851–0.937	<0.01
School Environmental project importance									
Low	1			1					
Median	1.060	1.031–1.090	<0.01	1.022	0.976–1.070	0.36	0.977	0.951–1.004	0.10
High	1.071	0.978–1.172	0.14	0.940	0.922–0.959	<0.01	0.965	0.846–1.101	0.60
Private school	1.213	1.175–1.253	<0.01	0.916	0.875–0.959	<0.01	1.020	0.974–1.068	0.41
Respiratory disease	1.070	1.001–1.144	0.05	0.944	0.879–1.014	0.11	1.011	0.947–1.081	0.74
Visits to a doctor in the last year	0.967	0.928–1.007	0.11	0.968	0.927–1.010	0.13	0.918	0.876–0.961	<0.01
Practice sports	0.975	0.925–1.027	0.34	1.007	0.982–1.032	0.60	0.918	0.875–0.964	<0.01

$e^{B}$ , coefficient exponential means prevalence rate ratio; 95%CI, 95% Confidence interval.

Lastly, awareness of air quality alerts twice a year was higher with increasing grade levels. Among 8-9th grade students, the prevalence ratio (PR) was 1.24 (95% CI: 0.99–1.54), and among 10-11th grade was 1.43 (95% CI: 1.43–1.81), while among students attending private schools was 1.57 (95% CI: 1.04–2.36). See Supplementary Table S7.

## 4 Discussion

To our knowledge, this is the first study in Colombia that evaluates the knowledge, attitudes, and practices of children and adolescents about air pollution and its potential health effects. Overall, the findings of this study showed that 81.1% of participants self-perceived as having low knowledge about air pollution. Other studies have reported a lower percentage of students (65%) who self-rated to have low knowledge about air pollution compared to our study (38).

In our study, children and adolescents had a limited knowledge of the air’s composition, only 31.1% recognized nitrogen as a component of clean air. Our finding is much lower than other studies conducted in Iran (58%), the UK (60%), Australia (53%), and Hong Kong (61%) (23, 28). The fact that many more children and adolescents perceive oxygen as a component of clean, unpolluted air regardless of its concentration, but do not recognize nitrogen as a component, can be attributed to educational approaches. In disciplines such as the sciences, it is possible to understand and verify the active role oxygen plays in a variety of natural ecosystem processes. As a gas that forms the typical mixture of air gases, nitrogen is generally referred to as an air pollutant due to the formation of nitrogen oxides and acids. In terms of ozone and the greenhouse effect, just 34% of participants understood ozone as an air component and 53% associated the greenhouse effect with pollution, compared to 63.7 and 65.3% of Chinese adolescents, respectively (38). These low results could also be explained because some concepts included in the knowledge component in Myers’ questionnaire (24) are related to atmospheric chemistry, which students in lower grades do not have in-depth knowledge of, and because the relationship for some components of the air may be confusing. For example, ozone often causes confusion based on its status as a ground-level pollutant but a protective gas in the stratosphere.

It is essential to address environmental issues in elementary and secondary schools to increase students’ knowledge (11, 17). Our study showed that a third of students had limited knowledge about air pollution and its sources; similar to prior research (24, 39). However, in a Spanish study, 65% of participants were knowledgeable of acid rain, whereas in our study, just 33% knew about it (39). This difference may be explained by the fact that in Europe, particularly in Spain, the problem of air pollution and its effects, specifically acid rain, have been front-page news for more than three decades in newspapers with a wide readership (40), while in our country, disclosure and denunciation of air pollution have just been made in the last decade, focusing especially on particulate matter, which is the pollutant that has increased the most in our region. According to WHO, the annual concentration of PM_2.5_ in the last decade for Barcelona, a city similar in urban aspects to Medellín, ranged between12 g/m^3^ and 18 g/m^3^, while for the Colombian city, it ranged between 20 g/m^3^ and 33 g/m^3^ (41).

In our study, more than 80% of participants associated air pollution as a cause of respiratory diseases, similar to what was found in various research studies (23, 24, 28, 38, 42). This can be explained by the fact that air pollutants are volatile, and as air enters the lungs, it is more easily correlated with pathologies affecting the respiratory tract system. Also, two-thirds of students in our study recognized that air pollution can cause adverse effects in pregnancy, probably due to the strong evidence between air pollution and adverse effects in pregnancy (5, 8, 43, 44), and because children associate polluted air with tobacco, which is strongly linked to adverse effects in pregnancy (45). However, we also acknowledge that children and adolescents may have considered that any outcome was possible, regardless of their actual knowledge about how air pollution is linked to pregnancy outcomes.

We found that students agreed that the strategy of having more education, more duties and taxes, and more legislation to reduce air pollution should be focused on society as a whole, but when measures touch on personal actions, it tends to decrease. In this sense, more than 60% of the participants considered that the payment of taxes should be taken over by industry and transportation companies rather than by citizens (Figure 3). Similar results were reported in Peru, Australia, and the UK (24, 25, 46), where students tend to agree that it is the responsibility of control entities or third parties to take action to reduce air pollution. Paradoxically, in terms of responsibility in caring for children and adolescents against the effects of air pollution, our study found that 66.3% of students think it corresponds to each citizen, followed by the government, industries, and the family. These results demonstrate how people are increasingly aware of their responsibility and role as citizens in reducing environmental air pollution (47). Less than 30% of students recognize healthcare staff and scientists as key actors in protecting children and adolescents. These findings are similar to other research (48).

It was observed that 38.8% of participants knew about air quality index, and only 15.6% of those who knew about it also consulted it. These findings are similar to others (49) and higher than the 22.6% found in US adolescents without asthma (27). About knowledge of air quality alerts twice a year in their municipalities, only 31% of the participants knew about them. This is similar to the knowledge among secondary students in Mexico (42) and higher than that of U.S adolescents (19%) (18).

Two hypotheses could explain this low knowledge score of air quality alerts. First, students might perceive air quality issues as a “problem of the other,” leading to a lack of interest in and distancing of the problem (42). Second, although environmental authorities implement various strategies to address environmental problems, such as the Integral Air Quality Management Plan (PIGECA), School Environmental Projects (PRAE), and scientific citizens through the SIATA app, these efforts are not well-articulated. They do not involve a continuous and permanent process of environmental education for citizens and schools, nor have they adequately characterized the target population to effectively communicate the health risks associated with air pollution and persuade people to adopt behaviors to reduce the risks associated with exposure (14, 50).

It has been shown that when these strategies are used in schools, they increase both knowledge and awareness among students (10, 17, 51). Nuria Castell and colleagues looked at children between 6 and 12 years old in several European cities. They used the “scientific citizens” approach and found that it helped kids develop scientific projects, learn about the environment, and come up with ways to improve the air quality in their cities. Even though the effects of these strategies on the population in Colombia and our region have not been studied, the results of our study may reflect a lack of communication between environmental authorities, education authorities, and schools, and therefore, the different strategies previously mentioned have little effect on people’s knowledge about air pollution and how it affects their health. Focusing on environmental education as a permanent and long-term strategy, starting with young children and adolescents, will help people develop values and attitudes that will allow them to live in better harmony with the environment.

Our research found that being female, studying at the eighth to eleventh-grade level, attending a private school, and attending institutions where the importance of the School Environmental Project is medium in its construction and implementation were associated with knowledge scores about air pollution. The finding of low knowledge among girls was consistent in the analyses conducted with the three proxy outcomes for particulate matter pollution. It has already been documented that they have less knowledge but have and are more committed to pro-environmental attitudes, which we also found in our study (16). The fact that students with higher degrees have spent more time participating in the school environmental project may help to explain these findings by increasing their knowledge of and improving their attitude toward the issues caused by air pollution (52, 53). However, the Myers’ et.al questionnaire included a knowledge component related to atmospheric chemistry concepts, which are more likely to be present in the curriculum at higher grade levels. Furthermore, alarmingly, we found that despite air pollution being an environmental issue exacerbated twice a year in the municipalities under study, this environmental problem was not prioritized in any of the eight school environmental projects. Moreover, knowledge of air quality alerts in the municipalities showed a dose–response effect based on grade level (16), with less knowledge among students in 6th – 7th grade.

As for how the private nature of the school affects knowledge, it has been shown that in private schools that best execute the School Environmental Project, students have better knowledge but not good environmental practices (16, 54). Our study found that although increasing knowledge is associated with studying in private schools, only 11.8% of students in these institutions perceived their knowledge of air pollution as high, very similar to students in public schools (13.6%). Additionally, this contrasts with the fact that none of the three environmental school projects in private schools ranked at the highest level of importance. Other socioeconomic factors could also explain this result. Students in private schools come from households with a better quality of life, greater access to technological resources, and higher levels of education among their parents (16, 18). This was reflected in the sources used to learn about air quality, which were very different, with much more learning from teachers and the internet in public schools, and more learning from parents or social networks in private schools. Therefore, it might not only be the schools, but also the general social environment.

Other factors that were associated with knowledge were having a respiratory disease, a high attitude score and concern toward air pollution and its consequences, which is similar to other studies (19, 23, 55, 56). Contrary to what was found in our study, a study in adolescents showed that having asthma did not result in differences in the level of awareness, knowledge of the impact of air pollution on their health, and having heard about air quality alerts (27). The concern of children and adolescents about the effects of air pollution is a driving force to get more informed, accept self-imposed individual restrictions, and take action to reduce the negative effects of air pollution, as long as their needs and expectations are reflected in risk communication messages (14).

Almost all students felt some degree of concern about air pollution, a much higher percentage than in Australia (73%) (25), the United Kingdom (87%) (24), and Peru (58%) (57). This positive attitude in favor of the environment is more evident among girls, a result similar to that reported by Catalán-Velásquez et al. (42) in Mexico. They found that girls have a greater pro-environmental attitude and tend to perceive more seriously the impact of air pollution on health, leading them to engage in pro-environmental practices (16).

On the other hand, studying in a private school and having a school environmental project with high importance were associated with lower attitudes scores toward actions to reduce air pollution. Students in private schools may not perceive the air pollution or poor air quality in their vicinity (home, school) due to the economic conditions in which they live (larger green areas and less crowding conditions). In addition, the private schools included in this study had a low or medium level of importance regarding their experience in implementing the School Environmental Project. Therefore, other factors such as economic resources, lower parental education levels (18), belief in state organizations (12), self-efficacy levels regarding their actions, and environmental awareness in the neighborhoods where students live could have contributed to this outcome more than the school itself (16).

In the majority of schools in our country, the School Environmental Project is an individual project that is implemented in a fragmented manner with little institutional support in terms of teachers or financial resources, and is carried out by one or a few science professors with the assistance of some students. We believe this is the reason why most students did not integrate environmental education with their environment, practices, or habits. The School Environmental Project would have a stronger impact when it connects activities in different areas of the learning process and is considered a permanent process of multidisciplinary, comprehensive, and strategic process in the training of students, and designed, planned, coordinated, and executed by the directives themselves.

Although our study reported high awareness and concern about air pollution, not always these pro-environmental attitudes are reflected in practices and behaviors, similar to other studies (38, 42, 57, 58), which have documented this gap in non-pro-environmental behaviors in a tourism context (59). Our results contradict Quian’s et al. (60) research, in which pro-environmental attitudes are associated with good practices and pro-environmental behavior. Personal protection practices against air pollution in our study were low compared to what was found in other studies (27). Rajper et al. (26) found that 68.2% of university and secondary Chinese students used face masks. In contrast, our study found that 73.6% of participants did not use face masks and did not take other preventive measures, such as reducing the opening of doors and windows (54.1%) or avoiding leaving the house due to air pollution (58.5%). These protective mechanisms are recommended by the environmental authority in the municipalities under study and are available in the SIATA app. However, 37.7% responded that they did not know about the air quality alert system. The poor implementation of personal protection measures against the effects of air pollution could be a consequence of ineffective communication that fails to increase environmental awareness (12), as well as of the poor pro-environmental attitude (38), since attitudes and perspectives play an important role in adopting a new health behavior (14).

Despite being aware of the dangers of daily exposure to poor air quality, little implementation of practices to prevent the deleterious effects of air pollution has been reported in adolescents (18). The low pro-environmental practices could also be explained by the limited evidence on the effectiveness and safety of individual-level interventions to reduce the health impacts of air pollution due to the few existing studies and the lack of methodological standardization in assessing the intervention and outcomes (61).

Regarding medical visits associated with lower pro-environmental practices, this could be explained by the fact that doctors rarely explain that respiratory diseases can also be caused by air pollution. In adolescents with asthma, doctors explained how to limit exposure to air pollution in 12.5% of cases compared to 2.1% in adolescents without asthma (27). These results contrast with what was found in adults with respiratory diseases, where there was an association with discussing it with a health professional (prevalence ratio = 4.88, 95% CI = 3.74, 6.37) (19). Hence, it is important to strengthening medical practice with the six strategies proposed by the WHO, given its key role in promotion and prevention actions to mitigate the effects of air pollution (5).

### 4.1 Strengths and limitations

The main strengths of our study are the sample size and sampling design, the standardized evaluation of the implementation experience of the School Environmental Project, the anonymity of the questionnaires to encourage students to answer truthfully and without feeling judged, and the strategy of analysis and knowledge translation that led to the co-development of educational materials with students and schools, and the creation of a website^2^ to promote, not only in the participating schools but in all schools in the region, the possibility of visualizing the results. Finally, we adhered to the STROBE reporting guide for observational studies.

Our study has some limitations. First, there are more updated surveys of knowledge and practices that evaluate the composition of clean air and its linked to atmospheric chemistry concepts, and its association with the knowledge about air pollution. However, we analyzed the knowledge about air pollution with three specific questions used in more recent studies, and the associated factors were similar and consistent between those studies and the knowledge scale we analyzed. Second, in the practices component, self-efficacy was not taken into account. If students do not believe that they can protect themselves, they might not change their practices. Third, the use of masks as a respiratory protection practice must be analyzed considering that there are potential cultural differences, and the COVID-19 pandemic may have changed how people approach the use of masks and their willingness to consider them as an intervention. Our study was conducted prior to COVID pandemic, and some of the studies we contrasted with our results were from other countries and post-pandemic. Fourth, the low number of included schools limits comparisons and inferences regarding the level of importance of the school environmental project. Fifth, we did not ask participants individually about their involvement and knowledge of the School Environmental Project, which turned out to be associated with their knowledge and attitudes. The School Environmental Project is a mandatory educational strategy implemented at the whole school level. Although some students participated more actively due to their interest in the subject, none of the eight schools identified air pollution as an environmental issue. Finally, we did not explore other individual practices in addition to those that are previously known, nor did we review the temporal nature of the same.

## 5 Conclusion

Students had a low score of knowledge and inadequate practices scores about air pollution; however, they have higher attitude scores towards alternative solutions that could be exploited to improve pro-environmental practices. Strengthening the School Environmental Project may improve students’ KAP. However, additional studies are warranted to determine the extent to which the School Environmental Project can improve KAP.

Given the global air pollution issue and the importance of this problem for public health, studies in other regions of the country and the world are required to identify and implement tailored concrete solutions for these population groups, and to educate not only the general community but also professional and trained healthcare personnel to recognize environmental exposures and the health effects associated with them.

## Data availability statement

The datasets presented in this study can be found in online repositories. The names of the repository/repositories and accession number(s) can be found at: https://figshare.com/ and can be accessed with doi: https://doi.org/10.6084/m9.figshare.25020563.

## Ethics statement

The studies involving humans were approved by Health Research Ethics of the Pontifical Bolivarian University. The studies were conducted in accordance with the local legislation and institutional requirements. Written informed consent for participation in this study was provided by the participants' legal guardians/next of kin.

## Author contributions

DM: Conceptualization, Formal analysis, Funding acquisition, Methodology, Project administration, Supervision, Writing – original draft, Writing – review & editing. NC: Data curation, Investigation, Writing – original draft, Writing – review & editing. VA: Data curation, Investigation, Writing – original draft, Writing – review & editing. PB: Data curation, Investigation, Writing – original draft, Writing – review & editing. MP: Data curation, Investigation, Visualization, Writing – original draft, Writing – review & editing. LO: Conceptualization, Methodology, Writing – original draft, Writing – review & editing. BM-O: Conceptualization, Resources, Validation, Writing – original draft, Writing – review & editing. JC: Conceptualization, Methodology, Validation, Writing – original draft, Writing – review & editing. LL: Formal analysis, Visualization, Writing – original draft, Writing – review & editing. ZR: Conceptualization, Resources, Supervision, Writing – original draft, Writing – review & editing.

## Footnotes

^1^ https://siata.gov.co/siata_nuevo/

^2^ https://www.escuelaenelmapa.com
