# Supplementary material for: Knowledge, attitudes and practices about air pollution and its health effects in 6th to 11th-grade students in Colombia: a cross-sectional study
Source: Front Public Health. 2024 Jun 19;12:1390780. doi: 10.3389/fpubh.2024.1390780 (PMC11221384; doi:10.3389/fpubh.2024.1390780)
Supplement: Supplementary file 1 [file Table_1.DOCX]

Supplementary Material

# Supplementary Data

The data underlying this study is available in https://figshare.com/ and can be accessed with <https://doi.org/10.6084/m9.figshare.25020563.v3>. The questionnaires used in this study in both English (Table 2) and Spanish (Table 3) versions can be accessed with <https://www.frontiersin.org/articles/10.3389/fpubh.2024.1390780/full#supplementary-material>

# Supplementary Figures and Tables

## Supplementary Figures


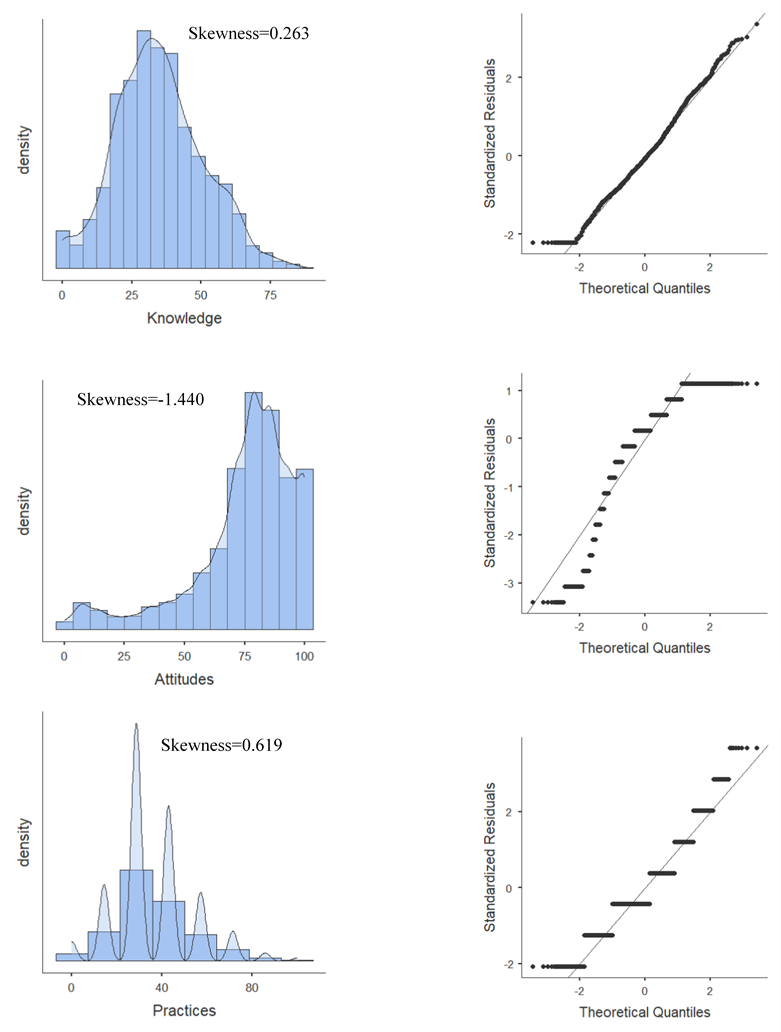


**Supplementary Figure 1.** Histogram and Q-Q plots to evaluate the distribution of probability of knowledge, attitudes and practices levels in elementary and high school students in Colombia, 2019–2020. A. Knowledge, B. Attitudes and C. Practices.


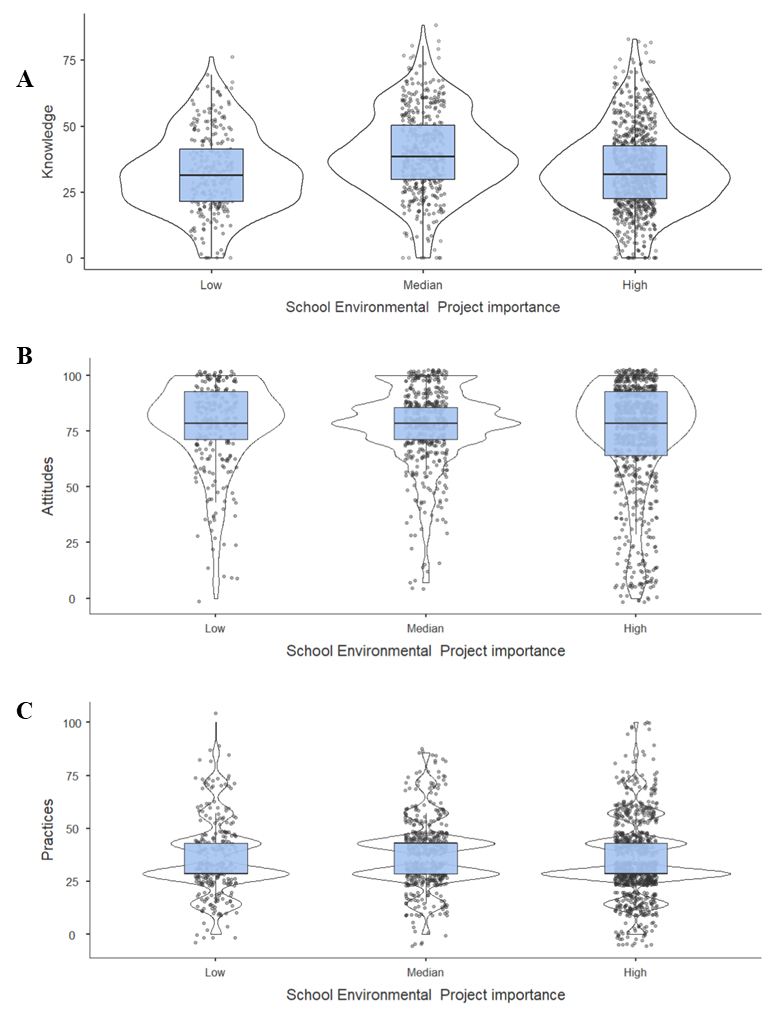


| Scale | Sig (Kruskal – Wallis) | Post-hoc |
| --- | --- | --- |
| Knowledge | <0.001 | Low<Median; High<Median |
| Attitudes | 0.231 | - |
| Practices | 0.001 | High<Median. |

**Supplementary Figure 2.** Distribution of knowledge, attitudes and practices levels in elementary and high school students according to the school environmental project importance. A. Knowledge, B. Attitudes and C. Practices.


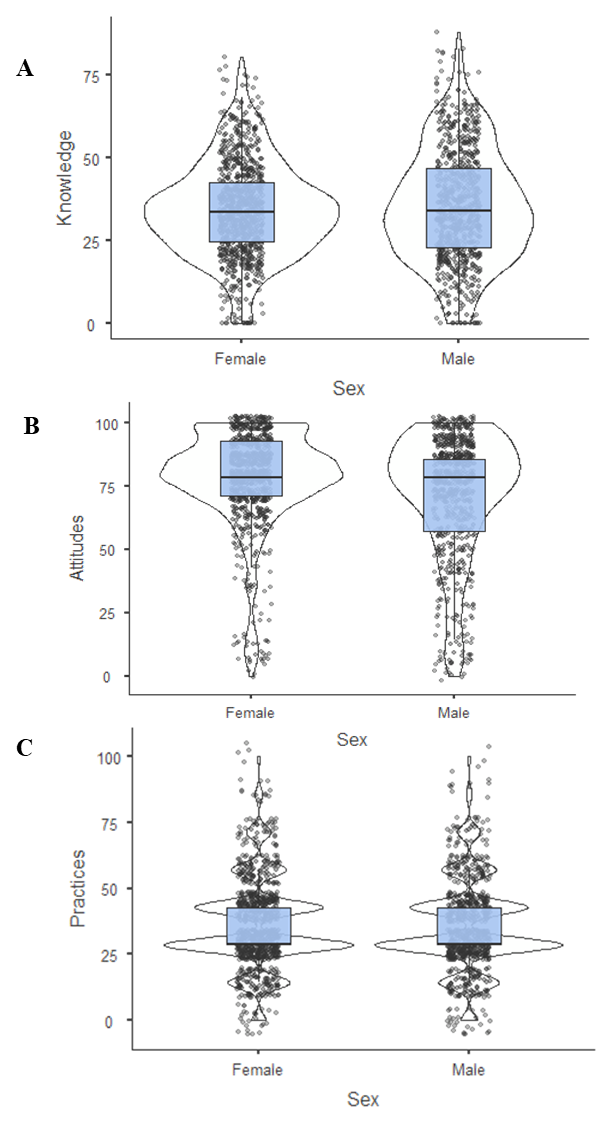


| Scale | Sig (Mann-Whitney U) |
| --- | --- |
| Knowledge | 0.338 |
| Attitudes | <0.001 |
| Practices | 0.559 |

**Supplementary Figure 3.** Distribution of knowledge, attitudes and practices levels in elementary and high school students according to sex. A. Knowledge, B. Attitudes and C. Practices.

**
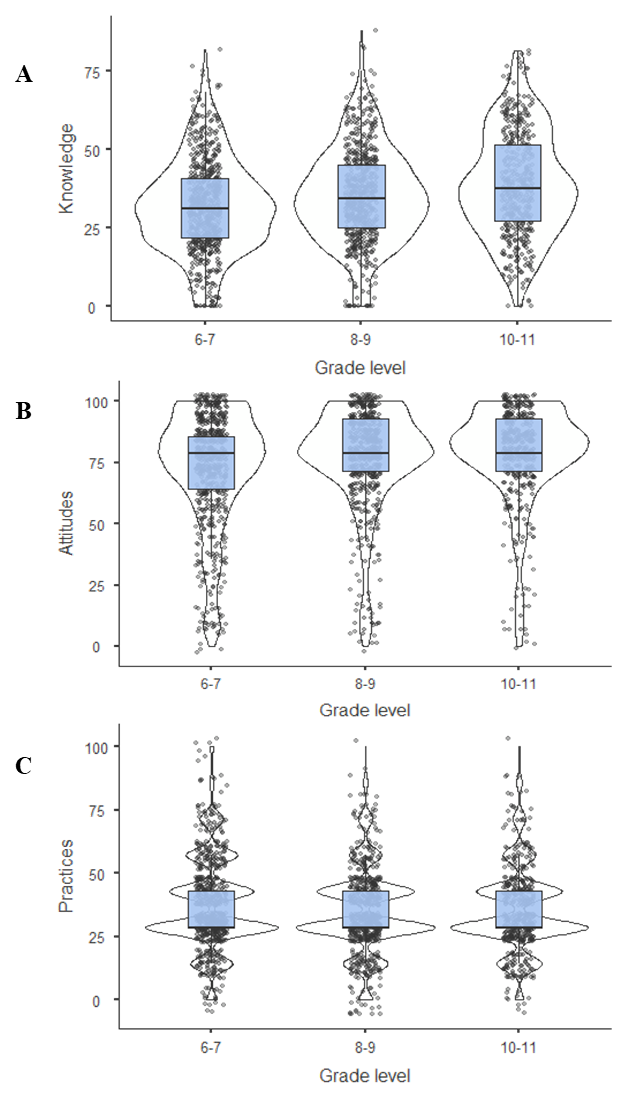
**

| Scale | Sig (Kruskal – Wallis) | Post-hoc |
| --- | --- | --- |
| Knowledge | <0.001 | 6-7<8-9; 6-7<10-11; 8-9<10-11 |
| Attitudes | <0.001 | 6-7<8-9; 6-7<10-11 |
| Practices | 0.014 | 6-7>8-9 |

**Supplementary Figure 4.** Distribution of knowledge, attitudes and practices levels in elementary and high school students according to the grade level. A. Knowledge, B. Attitudes and C. Practices.


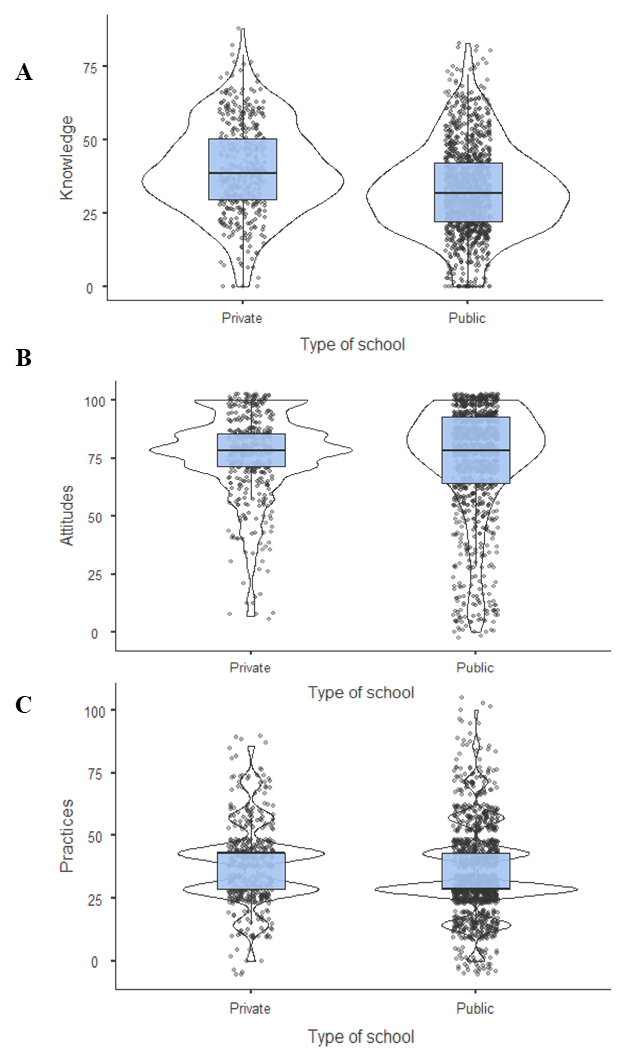


| Scale | Sig (Mann-Whitney U) |
| --- | --- |
| Knowledge | <0.001 |
| Attitudes | 0.781 |
| Practices | <0.001 |

**Supplementary Figure 5.** Distribution of knowledge, attitudes and practices levels in elementary and high school students according to the type of school. A. Knowledge, B. Attitudes and C. Practices.


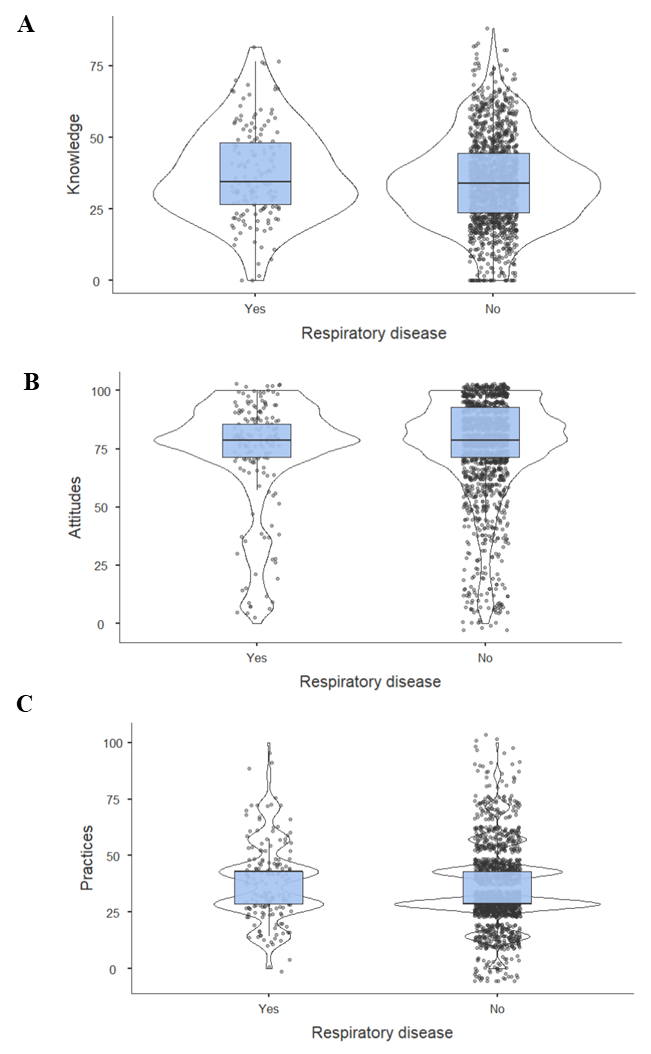


| Scale | Sig (Mann-Whitney U) |
| --- | --- |
| Knowledge | 0.042 |
| Attitudes | 0.498 |
| Practices | 0.098 |

**Supplementary Figure 6.** Distribution of knowledge, attitudes and practices levels in elementary and high school students according to have a respiratory disease. A. Knowledge, B. Attitudes and C. Practices.


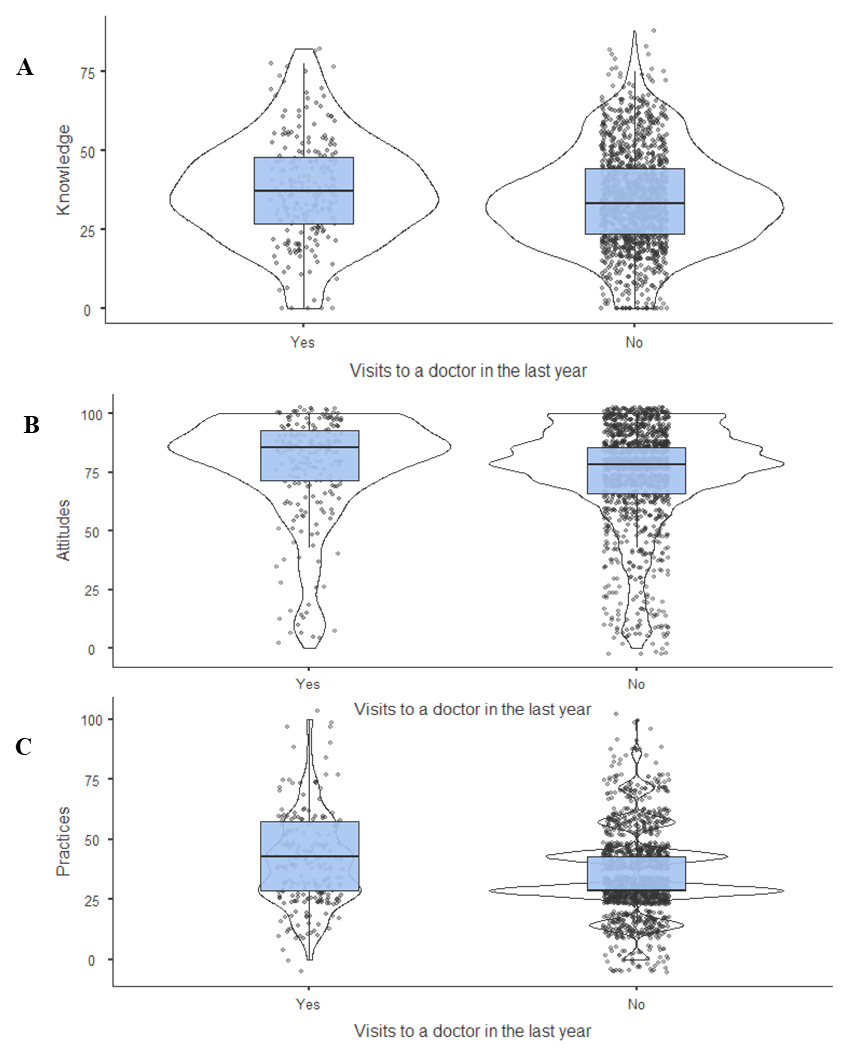


| Scale | Sig (Mann-Whitney U) |
| --- | --- |
| Knowledge | 0.003 |
| Attitudes | 0.008 |
| Practices | <0.001 |

**Supplementary Figure 7.** Distribution of knowledge, attitudes and practices levels in elementary and high school students according to visits to a doctor in the last year by respiratory symptoms . A. Knowledge, B. Attitudes and C. Practices.

**
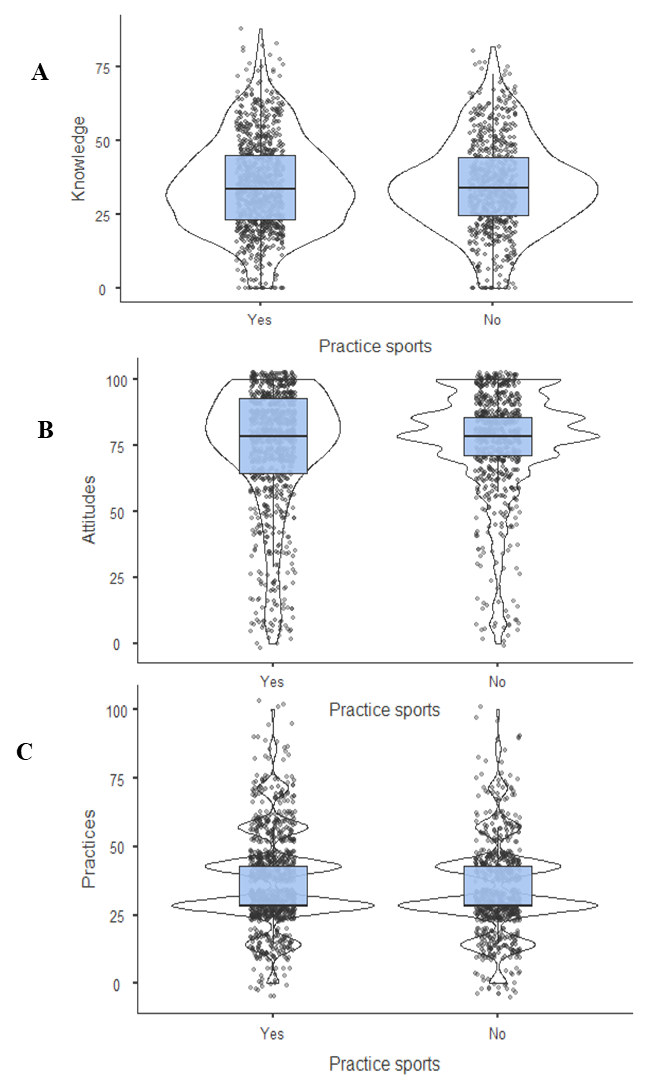
**

| Scale | Sig (Mann-Whitney U) |
| --- | --- |
| Knowledge | 0.997 |
| Attitudes | 0.206 |
| Practices | <0.001 |

**Supplementary Figure 8.** Distribution of knowledge, attitudes and practices levels in elementary and high school students according practicing sport. A. Knowledge, B. Attitudes and C. Practices.

## Supplementary Tables

**Supplementary Table S1.** Indicators for evaluating the importance of the School Environmental Project (PRAE) in environmental education of secondary school students in the KAP study

| **Dimension** | **Attribute PRAE** | **Indicator** | **Source** | **Score** | **Score interpretation** |
| --- | --- | --- | --- | --- | --- |
| General information | Not applicable | What is the main problem? | PRAE Coordinator | 0 | None |
|  |  |  |  | 1 | A defined theme |
|  |  |  |  | 3 | More than one theme |
|  |  | Definition of RESP objectives | Documents | 0 | It does not have |
|  |  |  |  | 1 | Under construction |
|  |  |  |  | 2 | Not in effect |
|  |  |  |  | 3 | Defined and updated |
|  |  | What have you achieved with the PRAE? | PRAE Coordinator | No score | Informative indicator |
|  |  | What is the impact of the PRAE in the I.E.? | PRAE Coordinator | No score | Informative indicator |
|  |  | What are the main difficulties encountered in the proper development of the RESP? | PRAE Coordinator Students focus group | No score | Informative indicator |
| Context analysis | Research | Which environmental diagnosis(s) were taken into account for the elaboration of the PRAE? | PRAE Coordinator | 0 | No diagnosis |
|  |  |  |  | 1 | Regional or national diagnosis |
|  |  |  |  | 2 | Diagnosis of educational institution |
|  |  |  |  | 3 | City environmental diagnosis |
|  | Research | Methodology for analysis of the environmental context of the institution and the community to define what problem the PRAE wanted to solve. | PRAE Coordinator | 0 | No context analysis was performed |
|  |  |  |  | 3 | Methodology for defining the problem is defined |
|  | Research | Process of prioritization of the environmental issues that were taken into account for the elaboration of the PRAE. | Documents | 0 | No process |
|  |  |  |  | 3 | It is defined through problem prioritization |
|  | Interdisciplinary | Participation of teachers in the identification of the problem on which the PRAE is focused. | PRAE Coordinator | 0 | Not involved |
|  |  |  |  | 1 | Natural sciences only |
|  |  |  |  | 2 | Natural sciences and other areas |
|  |  |  |  | 3 | All academic areas |
|  | Relevance | With whom he has shared his learning | Students focus group | 0 | They have not shared knowledge and experiences |
|  |  |  |  | 3 | With family, friends and neighbors |
|  | Relevance | What changes have been generated in their lives by participating in environmental care activities? | Students focus group | 0 | No changes have been generated |
|  |  |  |  | 3 | Changes in habits, attitudes, life project, others |
| Inter-institutional coordination | Community | How the community participated in the definition of the problem | PRAE Coordinator | 0 | Not invited |
|  |  |  |  | 1 | Invited but did not participate |
|  |  |  |  | 2 | Moderately |
|  |  |  |  | 3 | Actively |
|  | Interinstitutional | Accompaniment of authorities and experts in what phase of the PRAE? | PRAE Coordinator | 0 | They did not consult with other institutions or external experts. |
|  |  |  | Documents | 3 | In contextualization, problem identification, formulation, implementation, evaluation and monitoring |
|  | Interinstitutional | They have offered advice to other schools for the formulation of PRAE. | PRAE Coordinator | 0 | No |
|  |  |  | Students focus group | 3 | Yes |
|  | Community | Have given talks on environmental issues to other schools. | PRAE Coordinator | 0 | No |
|  |  |  | Students focus group | 3 | Yes |
|  | Community | Have offered technical assistance to the community to solve the environmental problem addressed by the PRAE. | PRAE Coordinator | 0 | No |
|  |  |  | Students focus group | 3 | Yes |
| School agreement | Curriculum with an environmental dimension | Direct inclusion of the environmental problem within the subjects of the different academic areas of the curriculum. | PRAE Coordinator | 0 | Not included in the curriculum |
|  |  |  |  | 1 | Yes, in the areas of natural and exact sciences. |
|  |  |  |  | 2 | Yes, in science and other areas. |
|  |  |  |  | 3 | Yes, in at least one academic unit in all areas. |
|  | Pedagogical-didactic strategies | Didactic activities that have been implemented to promote the School Environmental Project | PRAE Coordinator | 0 | No activity |
|  |  |  | Students focus group | 3 | The strategies used are as follows |
|  | Dialogue of knowledge | Who chooses what activities to do or what strategies to implement and how this is done? | PRAE Coordinator | No score | Informative indicator |
|  | Reflection | The RESP processes are systematically documented. | PRAE Coordinator | 0 | No |
|  |  |  |  | 3 | Yes |
|  | Reflection | How the PRAE is monitored | PRAE Coordinator | 0 | Trial and error/ No follow-up |
|  |  |  |  | 1 | Advice from external entities and experts |
|  |  |  |  | 3 | Systematic review of documentation, activities within the framework of the PRAE/ In meetings with teachers from different areas, analysis and feedback is provided. |
|  | Reflection | Considering the problematic axis of the PRAE, how has it changed since the implementation of the PRAE? | PRAE Coordinator | 0 | It is not known if it has improved |
|  |  |  |  | 1 | No improvement |
|  |  |  |  | 2 | Gradual improvement |
|  |  |  |  | 3 | Significant improvement |
| Participation processes | Not applicable | Total number of participants | Documents | 0 | ≤ 2 |
|  |  |  |  | 1 | 3-5 |
|  |  |  |  | 2 | 6-9 |
|  |  |  |  | 3 | ≥ 10 |
|  | Dialogue of knowledge | How the results of the PRAE are communicated at the institutional and community level | PRAE Coordinator | 0 | No communication |
|  |  |  |  | 1 | Teachers' meetings, parents' meetings, academic councils, academic events |
|  |  |  |  | 2 | IE website, networks or virtual communities |
|  |  |  |  | 3 | School communication media: radio station, newspapers, pedagogical days |
|  | Reflection | Communication and awareness-raising activities to promote the PRAE at institutional and community level involve | PRAE Coordinator | 0 | No |
|  |  |  |  | 1 | Sporadic activities open to the community |
|  |  |  |  | 2 | Periodic activities at school |
|  |  |  |  | 3 | Periodic activities in the IE and also involve the surrounding community and parents. |
|  | Dialogue of knowledge | Student participation in RESP activities | Students focus group | 0 | Do not get involved |
|  |  |  |  | 1 | They are involved through research projects |
|  |  |  |  | 2 | They carry out specific activities in the PRAE. |
|  |  |  |  | 3 | They are actively involved in the PRAE |
|  | Reflection | How students can become involved in the PRAE | PRAE Coordinator | 0 | No defined criteria |
|  |  |  |  | 1 | By order of the teachers |
|  |  |  |  | 2 | For school social service |
|  |  |  |  | 3 | On a voluntary basis |
|  | Interdisciplinary | Involvement of teachers in the implementation of the PRAE | PRAE Coordinator | 0 | Do not get involved |
|  |  |  |  | 1 | Natural sciences only |
|  |  |  |  | 2 | Natural sciences and other area(s) |
|  |  |  |  | 3 | All academic areas |
|  | Pedagogical-didactic strategies | Spaces have been created from the PRAE, such as forums, workshops, congresses or science fairs related only to environmental care. | PRAE Coordinator | 0 | No new |
|  |  |  |  | 2 | Yes sporadically |
|  |  |  |  | 3 | Yes on a periodic basis |
|  | Dialogue of knowledge | Who is the target audience for these spaces? | PRAE Coordinator | 0 | None |
|  |  |  |  | 1 | A specific audience |
|  |  |  |  | 3 | More than one target audience (students, guardians, community, teachers) |
|  | Administrative support | What is the administrative support to the PRAE? | PRAE Coordinator | 0 | No support |
|  |  |  |  | 2 | Support but limited |
|  |  |  |  | 3 | Full support to the PRAE |
|  | Community | How the community and parents are involved in PRAE activities | PRAE Coordinator | 0 | Do not participate |
|  |  |  |  | 2 | Sporadic participation |
|  |  |  |  | 3 | Actively participate |
| PRAE: School Environmental Project; EI: Educational Institution. | | | | | |

**Supplementary Table S2.** The total number of indicators assessed across the dimensions of the school environmental project. Colombia

| **Dimension** | **Total Indicators** | **Weight** | **Score** |
| --- | --- | --- | --- |
| Context analysis | 6 | 0.25 | 0 - 18 |
| Inter-institutional coordination | 5 | 0.25 | 0 - 15 |
| School agreement | 6 | 0.25 | 0 - 18 |
| Participation processes | 10 | 0.25 | 0 - 30 |
| **PRAE Evaluation** | **27** | **1.00** | **0 - 81** |

| **Importance categories - quartiles** | **School Environmental Project importance^1^** |
| --- | --- |
| ≤ 60 | Low importance |
| 61-70 | Median importance |
| ≥ 71 | High importance |
| ^1^ Quartiles obtained from the PRAE evaluation of the eight schools. | |

**Supplementary Table S3.** Number of students per type of school and level of importance of the school environmental project among elementary and high school students in Colombia, 2019–2020

| **School** | Private School  n= 3 | | | Public School  n=5 | | |
| --- | --- | --- | --- | --- | --- | --- |
|  | **School Environmental Project importance** | | | | | |
|  | Low | Median | High | Low | Median | High |
| School 1 | 0 | 153 | 0 | 0 | 0 | 0 |
| School 2 | 0 | 0 | 0 | 0 | 70 | 0 |
| School 3 | 0 | 258 | 0 | 0 | 0 | 0 |
| School 4 | 0 | 0 | 0 | 0 | 0 | 319 |
| School 5 | 0 | 0 | 0 | 0 | 0 | 335 |
| School 6 | 38 | 0 | 0 | 0 | 0 | 0 |
| School 7 | 0 | 0 | 0 | 245 | 0 | 0 |
| School 8 | 0 | 0 | 0 | 0 | 0 | 243 |

**Supplementary Table S4.** Distribution of knowledge of bad air quality alerts twice a year by grade level among elementary and high school students in Colombia, 2019–2020

| **Grade level n (%)** | **Knowledge of air quality alerts twice a year** | | | |
| --- | --- | --- | --- | --- |
|  | Completely known | Partially known | Not known at all | Total |
| 6 - 7 | 164 (26.7) | 278 (45.3) | 172 (28.0) | 614 |
| 8 – 9 | 182 (32.2) | 256 (45.3) | 127 (22.5) | 565 |
| 10 – 11 | 152 (34.9) | 204 (46.9) | 79 (18.2) | 435 |

**Supplementary Tables S5.** Descriptive statistics of knowledge, attitudes, and practices scores among elementary and high school students in Colombia, 2019–2020

| **Variable** | **Knowledge** | | | **Attitudes** | | | **Practices** | | |
| --- | --- | --- | --- | --- | --- | --- | --- | --- | --- |
|  | Median | Q_1_ | Q_3_ | Median | Q_1_ | Q_3_ | Median | Q_1_ | Q_3_ |
| **School Environmental Project importance** | | | | | | | | | |
| Low | 31.40 | 21.46 | 41.25 | 78.57 | 71.43 | 92.86 | 28.57 | 28.57 | 42.86 |
| Median | 38.47 | 29.80 | 50.25 | 78.57 | 71.43 | 85.71 | 42.86 | 28.57 | 42.86 |
| High | 31.73 | 22.47 | 42.42 | 78.57 | 64.29 | 92.86 | 28.57 | 28.57 | 42.86 |
| **Sex** |  |  |  |  |  |  |  |  |  |
| Female | 33.84 | 24.92 | 42.68 | 78.57 | 71.43 | 92.86 | 28.57 | 28.57 | 42.86 |
| Male | 33.88 | 22.81 | 46.89 | 78.57 | 57.14 | 85.71 | 28.57 | 28.57 | 42.86 |
| **Grade level** |  |  |  |  |  |  |  |  |  |
| 6-7 | 31.10 | 21.8 | 40.99 | 78.57 | 64.29 | 85.71 | 28.57 | 28.57 | 42.86 |
| 8-9 | 34.43 | 24.92 | 45.29 | 78.57 | 71.43 | 92.86 | 28.57 | 28.57 | 42.86 |
| 10-11 | 37.54 | 27.10 | 51.43 | 78.57 | 71.43 | 92.86 | 28.57 | 28.57 | 42.86 |
| **Private school** |  |  |  |  |  |  |  |  |  |
| Yes | 38.80 | 29.88 | 50.51 | 78.57 | 71.43 | 85.71 | 42.86 | 28.57 | 42.86 |
| No | 31.82 | 22.22 | 42.34 | 78.57 | 64.29 | 92.86 | 28.57 | 28.57 | 42.86 |
| **Respiratory disease** | |  |  |  |  |  |  |  |  |
| Yes | 34.60 | 26.30 | 48.27 | 78.57 | 71.43 | 85.71 | 42.86 | 28.57 | 42.86 |
| No | 33.84 | 23.74 | 44.36 | 78.57 | 71.43 | 92.86 | 28.57 | 28.57 | 42.86 |
| **Visits to a doctor in the last year** | | | | |  |  |  |  |  |
| Yes | 37.21 | 27.02 | 48.15 | 85.71 | 71.43 | 92.86 | 42.86 | 28.57 | 57.14 |
| No | 33.25 | 23.40 | 44.36 | 78.57 | 64.29 | 85.71 | 28.57 | 28.57 | 42.86 |
| **Practice sports** |  |  |  |  |  |  |  |  |  |
| Yes | 33.71 | 23.40 | 45.29 | 78.57 | 64.29 | 92.86 | 28.57 | 28.57 | 42.86 |
| No | 34.13 | 24.83 | 44.36 | 78.57 | 71.43 | 85.71 | 28.57 | 28.57 | 42.86 |

Q1: Percentile 25. Q3: Percentile 75

**Supplementary Tables S6.** Spearman correlation between the scores obtained in knowledge. attitudes. and practices among elementary and high school students in Colombia. 2019–2020.

| Score | | **Knowledge** | **Attitudes** | **Practices** |
| --- | --- | --- | --- | --- |
| **Knowledge** | Correlation | 1 |  |  |
|  | P-value | - |  |  |
|  | n | 1649 |  |  |
| **Attitudes** | Correlation | 0.246^**^ | 1 |  |
|  | P-value | <0.001 | - |  |
|  | n | 1645 | 1655 |  |
| **Practices** | Correlation | 0.254^**^ | 0.119^**^ | 1 |
|  | P-value | <0.001 | <0.001 | - |
|  | n | 1649 | 1655 | 1661 |
| ^**^The correlation is significant at the 0.01 level (two-tailed). | | | | |

**Supplementary Tables S7.** Factors associated with knowledge of air quality alerts. air quality index. and high self-perceived knowledge on air pollution among elementary and high school students.

| **Variable** | **Knowledge of air quality alerts twice a year** | | | **High self-perceived knowledge on air pollution** | | | **Knowledge of Air quality index** | | |
| --- | --- | --- | --- | --- | --- | --- | --- | --- | --- |
|  | **PR** | **95%CI** | **p-value** | **PR** | **95%CI** | **p-value** | **PR** | **95%CI** | **p-value** |
| Attitudes | 1.00 | 1.00-1.01 | 0.24 | 1.01 | 1.00-1.02 | 0.01 | 1.01 | 1.00-1.02 | 0.237 |
| Practices | 1.02 | 1.01-1.02 | < 0.01 | 1.02 | 1.01-1.03 | < 0.01 | 1.04 | 1.03-1.05 | < 0.01 |
| Female sex | 0.73 | 0.60-0.89 | 0.00 | 0.56 | 0.41-0.76 | < 0.01 | 0.55 | 0.34-0.87 | 0.012 |
| Grade level |  |  |  |  |  |  |  |  |  |
| 6 – 7 | 1 |  |  | 1 |  |  | 1 |  |  |
| 8 – 9 | 1.24 | 0.99-1.54 | 0.06 | 0.70 | 0.50-0.98 | 0.04 | 0.90 | 0.52-1.57 | 0.714 |
| 10 – 11 | 1.43 | 1.13-1.81 | 0.00 | 0.62 | 0.42-0.92 | 0.02 | 1.60 | 0.94-2.72 | 0.086 |
| School Environmental Project importance | | | |  |  |  |  |  |  |
| Low | 1 |  |  | 1 |  |  | 1 |  |  |
| Median | 0.90 | 0.60-1.36 | 0.63 | 0.86 | 0.48-1.51 | 0.59 | 0.81 | 0.31-2.12 | 0.672 |
| High | 1.16 | 0.86-1.56 | 0.34 | 0.60 | 0.40-0.92 | 0.02 | 1.27 | 0.67-2.42 | 0.457 |
| Private school | 1.57 | 1.04-2.37 | 0.03 | 0.69 | 0.39-1.23 | 0.21 | 1.57 | 0.61-4.08 | 0.351 |
| Respiratory disease (past or current) | 1.13 | 0.85-1.52 | 0.40 | 0.95 | 0.59-1.52 | 0.83 | 0.56 | 0.22-1.44 | 0.229 |
| Visits to a doctor in the last year by respiratory diseases | 1.15 | 0.89-1.47 | 0.28 | 1.61 | 1.12-2.32 | 0.01 | 1.12 | 0.61-2.03 | 0.717 |
| Practice sports | 1.07 | 0.88-1.30 | 0.49 | 1.09 | 0.79-1.50 | 0.61 | 1.34 | 0.82-2.21 | 0.244 |

PR: Prevalence ratio; CI: Confidence interval
