# Supplementary material for: Knowledge, attitudes and practices about air pollution and its health effects in 6th to 11th-grade students in Colombia: a cross-sectional study
Source: Front Public Health. 2024 Jun 19;12:1390780. doi: 10.3389/fpubh.2024.1390780 (PMC11221384; doi:10.3389/fpubh.2024.1390780)
Supplement: Supplementary file 2 [file Table_2.DOCX]

| **CODE** | | | |
| --- | --- | --- | --- |
|  |  |  |  |

Dear student,

This survey is not a test and no one from the school will know your answers, we simply want to know the knowledge, attitudes and practices that children and adolescents of the Aburrá Valley have regarding air pollution and its effects on health. We do this because at the end of the study we want to generate educational material with videos and other strategies, with those points that are unknown, so that all people learn a little more about this issue that affects us all.

If in any question you don't know the answer, don't worry, you can feel totally relaxed and mark the option **I don't know**, but please don't try to guess. Remember to complete all the questions and do not leave any blanks.

Thank you for your time!!!

**SOCIO-DEMOGRAPHIC CHARACTERISTICS**

***Mark with an X the option that most closely matches what you are being asked and answer the open-ended questions on the line.***

1. How old are you _______________
2. Sex

| Female |  |  | Male |  |
| --- | --- | --- | --- | --- |

1. Name of your educational institution ______________________________________________
2. Grade level ________________________________
3. In which municipality you live.

| Barbosa |  |  | Envigado |  |
| --- | --- | --- | --- | --- |
| Copacabana |  |  | Itagüí |  |
| Girardota |  |  | Sabaneta |  |
| Bello |  |  | The star |  |
| Medellín |  |  | Caldas |  |

1. Name of your neighborhood ______________________________
2. What do your parents do for a living? If you don't know, write I don't know:

- Madre __________________________________
- Padre __________________________________

1. Do you have any disease?

| Yes |  |  | No |  |
| --- | --- | --- | --- | --- |

8.1. If you said yes, tell us which one it is. ____________________________________________________________________________________________

1. In the past year, have you consulted a physician or emergency department for respiratory illnesses.

| Yes |  |  | No |  |
| --- | --- | --- | --- | --- |

9.1. If you said yes, tell us what happened to you:

____________________________________________________________________________________________

1. Do you practice any outdoor sports?

| Yes | No |
| --- | --- |

- 1. If you said yes, please indicate which sport and how many hours per week: ________________________________ _________________________________________

**Part 1**

***For each question, mark only one X for the option that most closely matches what you know.***

| Item | I am sure this is correct | I believe this is correct | I don't know about this | I believe this is incorrect | I am sure this is incorrect |
| --- | --- | --- | --- | --- | --- |
| 1. There is nitrogen in the clean, unpolluted air. |  |  |  |  |  |
| 1. There is ozone in the clean, unpolluted air. |  |  |  |  |  |
| 1. Methane in clean, unpolluted air |  |  |  |  |  |
| 1. There is water vapor in clean, unpolluted air. |  |  |  |  |  |
| 1. Hydrogen in clean, unpolluted air |  |  |  |  |  |
| 1. There is carbon dioxide in the clean, unpolluted air. |  |  |  |  |  |
| 1. Pollen in clean, unpolluted air |  |  |  |  |  |
| 1. There is oxygen in the clean, unpolluted air. |  |  |  |  |  |
| 1. There is carbon monoxide in clean, unpolluted air. |  |  |  |  |  |
| 1. There are other gases in clean, unpolluted air |  |  |  |  |  |

1. Which gas do you think is the one that exists in the greatest amount in clean air?

| Water vapor |  |  | Hydrogen |  |  | Oxygen |  |  | Carbon dioxide |  |  | Nitrogen |  |  | None of the above |  |
| --- | --- | --- | --- | --- | --- | --- | --- | --- | --- | --- | --- | --- | --- | --- | --- | --- |

***For each question, mark only one X for the option that most closely matches what you know.***

| Item | I am sure this is correct | I believe this is correct | I don't know about this | I believe this is incorrect | I am sure this is incorrect |
| --- | --- | --- | --- | --- | --- |
| 1. Air pollution occurs naturally, if there were no people in the world, the air would still be polluted. |  |  |  |  |  |
| 1. If the air smells good, it is not polluted. |  |  |  |  |  |
| 1. One factor in the contamination is the excess of a certain gas. |  |  |  |  |  |
| 1. Part of the air pollution is caused by animals |  |  |  |  |  |
| 1. Part of the air pollution is caused by plants |  |  |  |  |  |
| 1. If the air is clear, it is not polluted. |  |  |  |  |  |

| Item | I am sure this is correct | I believe this is correct | I don't know about this | I believe this is incorrect | I am sure this is incorrect |
| --- | --- | --- | --- | --- | --- |
| 1. Pollution influences plant growth |  |  |  |  |  |
| 1. If the air around us becomes more polluted, people who do not have asthma will start to have asthma. |  |  |  |  |  |
| 1. If the air around us becomes more polluted, people will get stomach diseases |  |  |  |  |  |
| 1. If the air around us becomes more polluted, people who already have asthma will have an even worse problem. |  |  |  |  |  |
| 1. If the air around us becomes more polluted, plants will not be able to produce seeds or reproduce as well. |  |  |  |  |  |
| 1. If the air around us becomes more polluted, people will get respiratory diseases |  |  |  |  |  |
| 1. If the air around us becomes more polluted, people will get brain diseases |  |  |  |  |  |
| 1. If pregnant women breathe polluted air, it is easier to harm their babies. |  |  |  |  |  |
| 1. If the air around us becomes more polluted, people will get heart disease |  |  |  |  |  |

| Item | I am sure this is correct | I believe this is correct | I don't know about this | I believe this is incorrect | I am sure this is incorrect |
| --- | --- | --- | --- | --- | --- |
| 1. There is more acid rain than there was before. |  |  |  |  |  |
| 1. There has to be some acid rain for plants and animals to survive. |  |  |  |  |  |
| 1. Some acid rain happens naturally, if there were no people in the world, acid rain would still exist. |  |  |  |  |  |

1. Who do you think should protect children and adolescents from air pollution? You can check more than one option, if you think so:

| The government |  |  | Every citizen |  |  |  |
| --- | --- | --- | --- | --- | --- | --- |
| Industries |  |  | Scientists |  |  |  |
| Doctors and nurses |  |  | Parents |  |  |  |
| Transportation companies |  |  | Another |  |  | Quién más_____________________________________ |

| Item | I am sure this is correct | I believe this is correct | I don't know about this | I believe this is incorrect | I am sure this is incorrect |
| --- | --- | --- | --- | --- | --- |
| 1. There is more greenhouse effect than there was before. |  |  |  |  |  |
| 1. There has to be some greenhouse effect for plants and animals to survive. |  |  |  |  |  |
| 1. Part of the greenhouse effect happens naturally, so if there were no people in the world, the greenhouse effect would still be |  |  |  |  |  |

1. How much do you think you know about air pollution?

| I know a lot |  |  | I know very little |  |  | I don't know anything |  |
| --- | --- | --- | --- | --- | --- | --- | --- |

**Part 2**

| Item | I strongly agree | I agree | I neither agree nor disagree | I disagree | I strongly disagree |
| --- | --- | --- | --- | --- | --- |
| 1. There should be more education about air pollution for me and my friends. |  |  |  |  |  |
| 1. Factories and transportation companies should do more to help stop air pollution |  |  |  |  |  |
| 1. We should all pay extra money to help stop air pollution |  |  |  |  |  |
| 1. Businesses should be taught more about air pollution |  |  |  |  |  |
| 1. There should be laws for me and my friends to do more to stop air pollution. |  |  |  |  |  |
| 1. We should all do more to help stop air pollution |  |  |  |  |  |
| 1. There should be laws to make factories and trucking companies do more to stop air pollution |  |  |  |  |  |
| 1. My friends and I should invest more money in air care. |  |  |  |  |  |
| 1. Everyone should be taught more about air pollution |  |  |  |  |  |
| 1. Industries and transportation companies should pay additional money to help stop air pollution |  |  |  |  |  |
| 1. My friends and I should be doing more to help stop air pollution. |  |  |  |  |  |
| 1. There should be laws to make everyone do more to stop air pollution |  |  |  |  |  |

1. How do you feel about air pollution?

| Very concerned |  |  | A little worried |  |  | I do not feel worried at all |  |
| --- | --- | --- | --- | --- | --- | --- | --- |

1. When someone approaches you about caring for the environment, how do you react (check one):

| I'm not really interested |  |
| --- | --- |
| I find it boring |  |
| I feel I am too young to help the environment. |  |
| I am more interested in the subject and ask how I can help. |  |
| I find out more about this topic from my parents, teachers or social networks. |  |

1. When you see buses or dump trucks spewing black smoke into the air, what do you think or do about it? _______________________

______________________________________________________________________________________________________

1. Tell us about different ways you help stop air pollution:

1. ___________________________________________
2. ___________________________________________
3. ___________________________________________
4. ___________________________________________
5. ___________________________________________
6. ___________________________________________

**Part 3**

1. Do you know what the air quality index (AQI) is?

| Fully known |  |  | More or less known |  |  | I do not know anything |  |
| --- | --- | --- | --- | --- | --- | --- | --- |

1. If you said you know something, do you check the SIATA air quality index daily?

| Yes |  |  | No |  |  | I don't know what SIATA is |  |
| --- | --- | --- | --- | --- | --- | --- | --- |

1. You use some of these sources to learn about air quality. You can check several

| I search on the internet |  |  | I ask a teacher |  |
| --- | --- | --- | --- | --- |
| I search in social networks |  |  | I ask my parents |  |
| I watch the news |  |  | I do nothing |  |

1. The Aburrá Valley, being surrounded by mountains and having a huge number of vehicles and motorcycles, has two times of the year in which the clouds do not let the air pollutants out and this causes bad air quality alerts. This is for you:

| Fully known |  |
| --- | --- |
| More or less known |  |
| I had no idea this was happening |  |

**
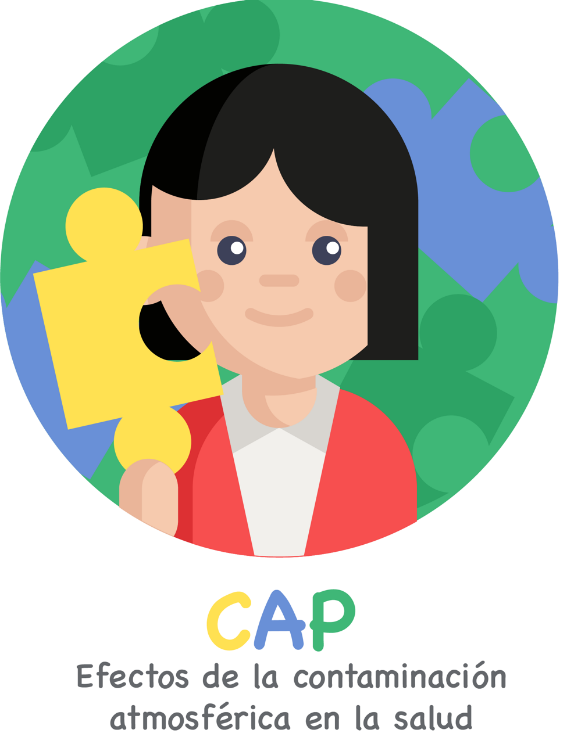
**

1. If you answered know at least something, can you tell us how you found out? You can check several options

| Internet |  |  | Friends |  |
| --- | --- | --- | --- | --- |
| Social networks |  |  | Teachers |  |
| News |  |  | My parents |  |
| Newspapers |  |  | College |  |

1. Has your school ever cancelled outdoor physical education classes due to air pollution problems?

| Sometimes |  |  | Rarely |  |  | Never |  |
| --- | --- | --- | --- | --- | --- | --- | --- |

1. Have you avoided leaving your home because of air pollution problems?

| Sometimes |  |  | Rarely |  |  | Never |  |
| --- | --- | --- | --- | --- | --- | --- | --- |

1. Have you used face masks to protect yourself from air pollution?

| Sometimes |  |  | Rarely |  |  | Never |  |
| --- | --- | --- | --- | --- | --- | --- | --- |

1. Does your home reduce the time it takes to open windows and doors when there are air pollution problems?

| Sometimes |  |  | Rarely |  |  | Never |  |
| --- | --- | --- | --- | --- | --- | --- | --- |

**THANK YOU FOR PARTICIPATING, YOUR INFORMATION IS VERY IMPORTANT FOR THE WHOLE CITY!**

Encuestador: ___________________________________

Survey date: ___________________ (DD - MM - YY)
