# Supplementary material for: Knowledge, attitudes and practices about air pollution and its health effects in 6th to 11th-grade students in Colombia: a cross-sectional study
Source: Front Public Health. 2024 Jun 19;12:1390780. doi: 10.3389/fpubh.2024.1390780 (PMC11221384; doi:10.3389/fpubh.2024.1390780)
Supplement: Supplementary file 3 [file Table_3.DOCX]

| **CÓDIGO** | | | |
| --- | --- | --- | --- |
|  |  |  |  |

Apreciado y apreciada estudiante,

Esta encuesta no es un examen ni nadie del colegio sabrá tus respuestas, simplemente queremos saber los conocimientos, actitudes y prácticas que los niños y adolescentes del valle de Aburrá tienen frente a la contaminación del aire y sus efectos en la salud. Esto lo hacemos porque al final del estudio queremos generar material educativo con videos y otras estrategias, con aquellos puntos que se desconocen, para que todas las personas aprendamos un poco más sobre este tema que nos afecta a todos.

Si en alguna pregunta no sabes la respuesta, no te preocupes, puedes sentirte totalmente tranquilo y marcar la opción **No sé**, pero por favor no trates de adivinar. Recuerda completar todas las preguntas y no dejar espacios en blanco.

Gracias por tu tiempo!!!

**CARACTERÍSTICAS SOCIODEMOGRÁFICAS**

***Marca con una X la opción que más se ajusta a lo que se te pregunta y responde las preguntas abiertas sobre la línea.***

1. Qué edad tienes _______________
2. Sexo

| Femenino |  |  | Masculino |  |
| --- | --- | --- | --- | --- |

1. Nombre de tu institución educativa ______________________________________________
2. Grado que cursas ________________________________
3. En qué municipio vives.

| Barbosa |  |  | Envigado |  |
| --- | --- | --- | --- | --- |
| Copacabana |  |  | Itagüí |  |
| Girardota |  |  | Sabaneta |  |
| Bello |  |  | La estrella |  |
| Medellín |  |  | Caldas |  |

1. Nombre de tu barrio ______________________________
2. ¿A qué se dedican tus padres? Si no lo sabes escribe no sé:

- Madre __________________________________
- Padre __________________________________

1. ¿Tienes alguna enfermedad?

| Si |  |  | No |  |
| --- | --- | --- | --- | --- |

8.1. Si dijiste que sí, cuéntanos cuál es. ____________________________________________________________________________________________

1. En el último año, has consultado al médico o a un servicio de urgencias por enfermedades respiratorias.

| Si |  |  | No |  |
| --- | --- | --- | --- | --- |

9.1. Si dijiste que sí, explícanos que te sucedió:

____________________________________________________________________________________________

1. ¿Practicas algún deporte al aire libre?

| Si | No |
| --- | --- |

- 1. Si dijiste que sí, indica cuál deporte y cuantas horas a la semana: ________________________________ _________________________________________

**Parte 1**

***Para cada pregunta marca solo una X la opción que más se ajusta a lo que sabes***

| Pregunta | Estoy seguro de que esto es correcto | Creo que esto es correcto | No sé sobre esto | Creo que esto es incorrecto | Estoy seguro de que esto es incorrecto |
| --- | --- | --- | --- | --- | --- |
| 1. Hay nitrógeno en el aire limpio y no contaminado |  |  |  |  |  |
| 1. Hay ozono en el aire limpio y no contaminado |  |  |  |  |  |
| 1. Hay metano en el aire limpio y no contaminado |  |  |  |  |  |
| 1. Hay vapor de agua en el aire limpio y no contaminado |  |  |  |  |  |
| 1. Hay hidrógeno en el aire limpio y no contaminado |  |  |  |  |  |
| 1. Hay dióxido de carbono en el aire limpio y no contaminado |  |  |  |  |  |
| 1. Hay polen en el aire limpio y no contaminado |  |  |  |  |  |
| 1. Hay oxígeno en el aire limpio y no contaminado |  |  |  |  |  |
| 1. Hay monóxido de carbono en el aire limpio y no contaminado |  |  |  |  |  |
| 1. Hay otros gases en el aire limpio y no contaminado |  |  |  |  |  |

1. ¿Cuál crees que es el gas que existe en mayor cantidad en el aire limpio? *marca solo uno*:

| Vapor de agua |  |  | Hidrogeno |  |  | Oxigeno |  |  | Dióxido de carbono |  |  | Nitrógeno |  |  | Ninguna de las anteriores |  |
| --- | --- | --- | --- | --- | --- | --- | --- | --- | --- | --- | --- | --- | --- | --- | --- | --- |

***Para cada pregunta marca solo una X la opción que más se ajusta a lo que sabes***

| Pregunta | Estoy seguro de que esto es correcto | Creo que esto es correcto | No sé sobre esto | Creo que esto es incorrecto | Estoy seguro de que esto es incorrecto |
| --- | --- | --- | --- | --- | --- |
| 1. La contaminación del aire ocurre naturalmente, si no existieran personas en el mundo, el aire aún estaría contaminado |  |  |  |  |  |
| 1. Si el aire huele bien, no está contaminado |  |  |  |  |  |
| 1. Un factor de la contaminación es el exceso de algún gas |  |  |  |  |  |
| 1. Parte de la contaminación del aire es causada por animales |  |  |  |  |  |
| 1. Parte de la contaminación del aire es causada por las plantas |  |  |  |  |  |
| 1. Si el aire se ve claro, no está contaminado |  |  |  |  |  |

| Pregunta | Estoy seguro de que esto es correcto | Creo que esto es correcto | No sé sobre esto | Creo que esto es incorrecto | Estoy seguro de que esto es incorrecto |
| --- | --- | --- | --- | --- | --- |
| 1. La contaminación influencia en el crecimiento de las plantas |  |  |  |  |  |
| 1. Si el aire que nos rodea se contamina más, las personas que no tienen asma empezarán a tener asma |  |  |  |  |  |
| 1. Si el aire que nos rodea se contamina más, las personas tendrán enfermedades del estómago |  |  |  |  |  |
| 1. Si el aire que nos rodea se contamina más, en las personas que ya tienen asma su problema se agravará |  |  |  |  |  |
| 1. Si el aire que nos rodea se contamina más, las plantas no serán capaces de producir semillas ni reproducirse tan bien |  |  |  |  |  |
| 1. Si el aire que nos rodea se contamina más, las personas tendrán enfermedades respiratorias |  |  |  |  |  |
| 1. Si el aire que nos rodea se contamina más, las personas tendrán enfermedades del cerebro |  |  |  |  |  |
| 1. Si las mujeres embarazadas respiran aire contaminado, se produce más fácil daño en sus bebes |  |  |  |  |  |
| 1. Si el aire que nos rodea se contamina más, las personas tendrán enfermedades del corazón |  |  |  |  |  |

| Pregunta | Estoy seguro de que esto es correcto | Creo que esto es correcto | No sé sobre esto | Creo que esto es incorrecto | Estoy seguro de que esto es incorrecto |
| --- | --- | --- | --- | --- | --- |
| 1. Hay más lluvia ácida de lo que había antes. |  |  |  |  |  |
| 1. Tiene que haber algo de lluvia ácida para que las plantas y los animales sobrevivan. |  |  |  |  |  |
| 1. Parte de la lluvia ácida sucede naturalmente, si no existieran personas en el mundo, la lluvia acida aún existiría |  |  |  |  |  |

1. ¿Quiénes crees que deberían proteger a los niños y adolescentes de la contaminación del aire? Puedes marcar más de una opción, si así lo consideras:

| El gobierno |  |  | Cada ciudadano |  |  |  |
| --- | --- | --- | --- | --- | --- | --- |
| Las industrias |  |  | Los científicos |  |  |  |
| Los médicos y enfermeras |  |  | Los padres de familia |  |  |  |
| Las empresas de transporte |  |  | Otro |  |  | Quién más_____________________________________ |

| Pregunta | Estoy seguro de que esto es correcto | Creo que esto es correcto | No sé sobre esto | Creo que esto es incorrecto | Estoy seguro de que esto es incorrecto |
| --- | --- | --- | --- | --- | --- |
| 1. Hay más efecto invernadero del que había antes. |  |  |  |  |  |
| 1. Tiene que haber algo de efecto invernadero para que las plantas y los animales sobrevivan. |  |  |  |  |  |
| 1. Parte del efecto invernadero sucede naturalmente, por lo que si no existieran personas en el mundo, el efecto invernadero aún estaría |  |  |  |  |  |

1. ¿Cuánto crees que sabes sobre la contaminación del aire?

| Sé Mucho |  |  | Sé Poquito |  |  | No sé nada en absoluto |  |
| --- | --- | --- | --- | --- | --- | --- | --- |

**Parte 2**

| Pregunta | Estoy muy de acuerdo | Estoy de acuerdo | No estoy de acuerdo ni en desacuerdo | Estoy en desacuerdo | Estoy muy en desacuerdo |
| --- | --- | --- | --- | --- | --- |
| 1. Debería haber más educación sobre la contaminación del aire para mí y mis amigos |  |  |  |  |  |
| 1. Las fábricas y las empresas de transporte deberían hacer más para ayudar a detener la contaminación del aire |  |  |  |  |  |
| 1. Todos deberíamos pagar plata adicional para ayudar a detener la contaminación del aire |  |  |  |  |  |
| 1. A las empresas les deberían enseñar más sobre la contaminación del aire |  |  |  |  |  |
| 1. Deberían haber leyes para que mis amigos y yo hagamos más para detener la contaminación del aire |  |  |  |  |  |
| 1. Todos deberíamos hacer más para ayudar a parar la contaminación del aire |  |  |  |  |  |
| 1. Debería haber leyes para hacer que las fábricas y las empresas de transporte hagan más para parar la contaminación del aire |  |  |  |  |  |
| 1. Mis amigos y yo deberíamos invertir más dinero en el cuidado del aire |  |  |  |  |  |
| 1. A todos les deberían enseñar más sobre la contaminación del aire |  |  |  |  |  |
| 1. Las industrias y las empresas de transporte deberían pagar dinero adicional para ayudar a detener la contaminación del aire |  |  |  |  |  |
| 1. Mis amigos y yo deberíamos hacer más para ayudar a parar la contaminación del aire |  |  |  |  |  |
| 1. Debería haber leyes para hacer que todos hagan más para detener la contaminación del aire |  |  |  |  |  |

1. ¿Qué sientes acerca de la contaminación del aire?

| Muy preocupado |  |  | Un poco preocupado |  |  | No me siento preocupado en absoluto |  |
| --- | --- | --- | --- | --- | --- | --- | --- |

1. Cuando alguien se acerca a ti para hablarte sobre el cuidado del medio ambiente, cómo es tú reacción (marca una):

| La verdad no me interesa |  |
| --- | --- |
| Me parece aburridor |  |
| Siento que soy muy joven para ayudar al medio ambiente |  |
| Me intereso más por el tema y pregunto cómo puedo ayudar |  |
| Averiguo más sobre ese tema con mis padres, profesores o redes sociales |  |

1. Cuando ves que buses o volquetas echan humo negro al aire, tú qué piensas o haces frente a eso: _______________________

______________________________________________________________________________________________________

1. Cuéntanos distintas formas en las que tu ayudas a parar la contaminación del aire:

1. ___________________________________________
2. ___________________________________________
3. ___________________________________________
4. ___________________________________________
5. ___________________________________________
6. ___________________________________________

**Parte 3**

1. ¿Sabes qué es el índice de calidad del aire (ICA)?

| Totalmente conocido |  |  | Más o menos conocido |  |  | No sé nada |  |
| --- | --- | --- | --- | --- | --- | --- | --- |

1. Si dijiste que sabías algo, ¿verificas en el SIATA el índice de calidad del aire diariamente?

| Si |  |  | No |  |  | No sé qué es SIATA |  |
| --- | --- | --- | --- | --- | --- | --- | --- |

1. Utilizas algunas de estas fuentes para aprender sobre la calidad del aire. Puedes marcar varias

| Busco en internet |  |  | Le pregunto a un profesor |  |
| --- | --- | --- | --- | --- |
| Busco en redes sociales |  |  | Le pregunto a mis padres |  |
| Veo las noticias |  |  | No hago nada |  |

1. El Valle de Aburrá por estar rodeado de montañas y por tener una enorme cantidad de vehículos y motos, tiene dos épocas del año en el que las nubes no dejan que los contaminantes atmosféricos salgan y esto provoca alertas por mala calidad del aire. Esto para ti es:

| Totalmente conocido |  |
| --- | --- |
| Más o menos conocido |  |
| No tenía ni idea que esto pasaba |  |

**
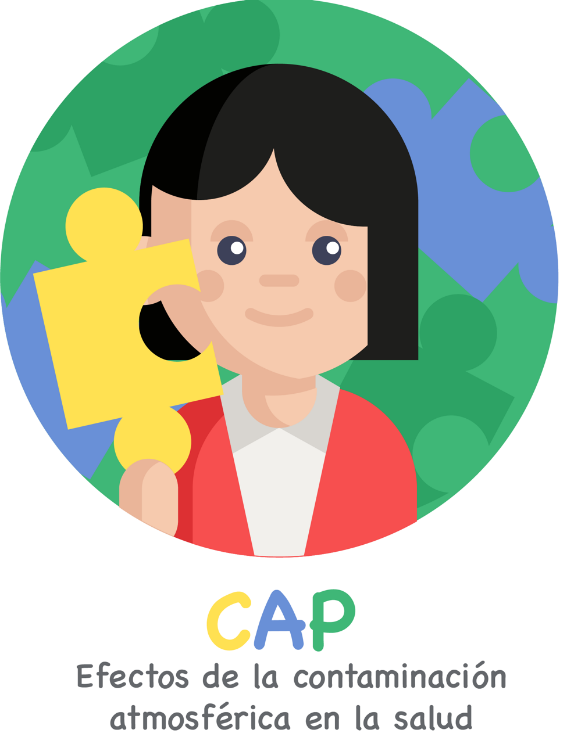
**

1. Si respondiste conocer al menos algo, ¿nos puedes contar como te enteraste? Puede marcar varias opciones

| Internet |  |  | Amigos |  |
| --- | --- | --- | --- | --- |
| Redes sociales |  |  | Profesores |  |
| Noticieros |  |  | Mis padres |  |
| Periódicos |  |  | Colegio |  |

1. ¿En tu colegio alguna vez cancelaron las clases de educación física al aire libre por problemas de contaminación en el aire?

| Algunas veces |  |  | Rara vez |  |  | Nunca |  |
| --- | --- | --- | --- | --- | --- | --- | --- |

1. ¿Has evitado salir de tu casa por problemas de contaminación en al aire?

| Algunas veces |  |  | Rara vez |  |  | Nunca |  |
| --- | --- | --- | --- | --- | --- | --- | --- |

1. ¿Has usado mascarillas para protegerte de la contaminación en el aire?

| Algunas veces |  |  | Rara vez |  |  | Nunca |  |
| --- | --- | --- | --- | --- | --- | --- | --- |

1. ¿En tu casa se reduce el tiempo de apertura de ventanas y puertas cuando hay problemas de contaminación en el aire?

| Algunas veces |  |  | Rara vez |  |  | Nunca |  |
| --- | --- | --- | --- | --- | --- | --- | --- |

**¡GRACIAS POR PARTICIPAR, TU INFORMACIÓN ES MUY IMPORTANTE PARA TODA LA CIUDAD!**

Encuestador: ___________________________________

Fecha encuesta: ___________________ (DD – MM – AA)
